# Low-Temperature Graphene-Based Paste for Large-Area Carbon Perovskite Solar Cells

**DOI:** 10.1021/acsami.1c02626

**Published:** 2021-05-10

**Authors:** Paolo Mariani, Leyla Najafi, Gabriele Bianca, Marilena Isabella Zappia, Luca Gabatel, Antonio Agresti, Sara Pescetelli, Aldo Di Carlo, Sebastiano Bellani, Francesco Bonaccorso

**Affiliations:** † CHOSE—Centre for Hybird and Organic Solar Energy, University of Rome Tor Vergata, Via del Politecnico 1, 00133 Rome, Italy; ‡ BeDimensional S.p.A., Via Lungotorrente Secca 3D, 16163 Genova, Italy; § Graphene Labs, Istituto Italiano di Tecnologia, Via Morego 30, 16163 Genova, Italy; ∥ Dipartimento di Chimica e Chimica Industriale, Università degli Studi di Genova, Via Dodecaneso 31, 16146 Genoa, Italy; ⊥ Department of Physics, University of Calabria, Via P. Bucci cubo 31/C, 87036 Rende, Cosenza, Italy; # ISM-CNR, Istituto di Struttura della Materia, Consiglio Nazionale delle Ricerche, Via del Fosso del Cavaliere 100, 00133 Rome, Italy

**Keywords:** perovskite solar cells, carbon, graphene, paintable, solution processing, large-area, scalability, metallization

## Abstract

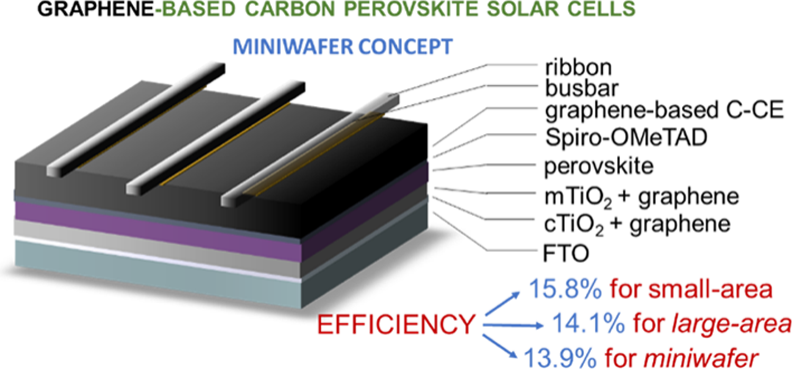

Carbon perovskite solar cells (C-PSCs), using carbon-based counter electrodes (C-CEs), promise to mitigate instability issues while providing solution-processed and low-cost device configurations. In this work, we report the fabrication and characterization of efficient paintable C-PSCs obtained by depositing a low-temperature-processed graphene-based carbon paste atop prototypical mesoscopic and planar n–i–p structures. Small-area (0.09 cm^2^) mesoscopic C-PSCs reach a power conversion efficiency (PCE) of 15.81% while showing an improved thermal stability under the ISOS-D-2 protocol compared to the reference devices based on Au CEs. The proposed graphene-based C-CEs are applied to large-area (1 cm^2^) mesoscopic devices and low-temperature-processed planar n–i–p devices, reaching PCEs of 13.85 and 14.06%, respectively. To the best of our knowledge, these PCE values are among the highest reported for large-area C-PSCs in the absence of back-contact metallization or additional stacked conductive components or a thermally evaporated barrier layer between the charge-transporting layer and the C-CE (strategies commonly used for the record-high efficiency C-PSCs). In addition, we report a proof-of-concept of metallized miniwafer-like area C-PSCs (substrate area = 6.76 cm^2^, aperture area = 4.00 cm^2^), reaching a PCE on active area of 13.86% and a record-high PCE on aperture area of 12.10%, proving the metallization compatibility with our C-PSCs. Monolithic wafer-like area C-PSCs can be feasible all-solution-processed configurations, more reliable than prototypical perovskite solar (mini)modules based on the serial connection of subcells, since they mitigate hysteresis-induced performance losses and hot-spot-induced irreversible material damage caused by reverse biases.

## Introduction

Perovskite solar cells (PSCs) are continuously attracting extensive attention among the photovoltaic (PV) technologies due to their low-cost solution processability^1^ accompanied by outstanding performances, which reached certified power conversion efficiencies (PCEs) above 25% (record-high value of 25.5%).^2−4^ These values are strongly competitive with the PCEs of monocrystalline and heterojunction (HJT) silicon solar cells (26.1 and 26.7%, respectively)^2^ and overpass certified ones of mass-affordable thin-film PV technologies, that is, copper indium gallium selenide and CdTe solar cells (23.4 and 22.1%, respectively).^2^ Even more, perovskite-based tandem configurations, especially perovskite-silicon tandem solar cells, have achieved a certified PCE of 29.1%, thus enabling cost-effective tandem configurations for next-generation PV industry.^5^ Nevertheless, the commercialization of the most efficient PSC forms is facing technical barriers,^6,7^ which mainly relates to the following: (1) the instability of the photoactive perovskites^8,9^ and charge-transporting layers (CTLs)^9^ and (2) the use of expensive, vacuum thermally evaporated noble metal-based back counter electrodes (CEs), for example, gold and silver.^10^ The chemical reactions between the perovskite layer and the CE through the migration of iodine species or metal atoms are also further causes of material instability in assembled devices.^11,12^ Therefore, the research community is struggling to design novel efficient and stable perovskites, CTLs, interlayers, and metal-free CEs, which mitigate the above issues while providing cost-effective, large-area processability.^1^ It is noteworthy that pressure-tight polymer (polyisobutylene)/glass stack encapsulation has been recently reported to prevent moisture intake while suppressing the outgassing of perovskite decomposition products.^13^ Consequently, the decomposition reaction for a prototypical triple cation perovskite [Cs_0.05_FA_0.8_MA_0.15_Pb(I_0.85_Br_0.15_)_3_] was blocked, allowing the PSCs to pass the International Electrotechnical Commission (IEC) 61215:2016 Damp Heat and Humidity Freeze tests. Prospectively, if an appropriate encapsulation would stabilize PSCs, the replacement of noble metal-based CEs would acquire even more importance to achieve an estimated levelized cost of energy (LCOE) of perovskite solar panels of less than 5 US cents kW h^–1^.^10,14^ Such a LCOE would be comparable or even inferior to the ones of commercially available silicon and thin-film-based solar PVs^10,14^ and competitive with the LCOEs of fossil fuels.^15^ In this context, carbon-based CEs (C-CEs) emerged as low-cost printable alternatives to Au- and Ag-based ones, leading to the so-called carbon-based PSCs (C-PSCs).^16,17^ Besides, the C-PSCs have the potential to minimize the CO_2_ footprint of PSC materials and manufacturing processes,^17,18^ paving the way for next-generation solar cells with low environmental harm.

Three types of C-PSCs have been classified, namely, mesoporous,^19,20^ embedment,^21,22^ and paintable C-PSCs.^23,24^ In the former, porous carbon electrodes are first deposited, and the perovskite precursor solution is infiltrated inside.^19,20^ Alternatively, the porous carbon electrode can be deposited onto a perovskite precursor (e.g., PbI_2_), followed by the conversion of the precursor to perovskite by infiltrating a reaction solution, leading to the so-called embedment C-PSCs.^21,22^ In paintable C-PSCs, the carbon electrode is directly deposited onto the perovskite layer, or the hole-transporting layer (HTL), or the electron-transporting layer (ETL) depending on the device configuration (i.e., CTL-free devices, n–i–p and p–i–n configurations, respectively).^23,24^ The main acclaimed advantages of C-CEs are as follows:^16,17,25^ (1) low cost, which, however, strongly depends on the type of the carbon materials. The hole-extraction properties of C-CEs can also eliminate the use of (expensive) HTLs required for noble metal-based CEs;^23,25,26^ (2) chemical inertness to halide ions, which eliminates the corrosion of metallic CEs;^9^ and (3) hydrophobic characteristics, which intrinsically limit the intake of moisture.^27^ These features are considered a breakthrough for the reduction of the LCOE of the current PSC technology.^28^ Unfortunately, the PCEs of C-PSCs still lag behind those of the most efficient Au-based PSCs.^17^ Furthermore, several C-PSCs with PCEs higher than 15% are often based on (doped) carbon nanotubes, which have been used as a hydrophobic additive in the perovskite layer,^29^ highly conductive and hole-extracting CE materials,^30,31^ and interlayers electrically connecting the perovskite to the CEs.^32,33^ Regrettably, the cost of solar-grade carbon nanotubes, including single-/double-walled ones, can be even superior to the one of noble metals.^34^ The highest PCE of 19.2% has been reached by “paintable-like” C-PSCs,^35^ in which the C-CE was realized through the hot-press transfer method.^35−37^ Although this method is compatible with high-throughput roll-to-roll manufacturing and/or laminating processes,^35,36,38^ its precise control over a large area has just been reported in 1 cm^2^ PSCs using a conductive (sheet resistance *R*_sheet_ < 1 Ω sq^–1^) graphite paper or aluminum foil as the substrate for the C-CEs (PCE = 17.4 and 15.41%).^39^ Meanwhile, perovskite solar modules (PSMs) based on hot-press transferred C-CEs have not been tested yet. On the contrary, printed C-PSCs have been upscaled into large-area configurations and PSMs^20,40−43^ and even into solar farms (up to 7 m^2^ area).^40,42^ In these circumstances, both screen printing^40^ and mechanical scribing^44^ have been proposed as methods to pattern the C-CEs for the serial interconnection of the cells in prototypical PSMs.^43,45^ Meanwhile, the use of advanced HTLs, such as poly(3-hexylthiophene-2,5-diyl) (P3HT)/graphene composite, enabled paintable C-PSCs to reach a record-high PCE of 18.2% (certified value up to 17.8%).^46^ Recent reviews on C-PSCs summarized the origin of their performance loss compared to state-of-the-art devices,^16,47^ which is consensually attributed to the insufficient charge-selective properties of the C-CEs, as well as to charge recombination processes at the back interface (i.e., perovskite/CE or CTL/CE).^17^ The engineering of perovskite, CE, and interlayer represents a common strategy to mitigate the current C-PSC limitations.^17^ Nevertheless, paintable C-PSCs based on low-temperature-processed CEs are currently considered convenient configurations to close the PCE gap between C-PSC and noble-metal ones.^17^ In particular, the knowledge used for the fabrication of the state-of-the-art PSCs is directly transferable into such C-PSCs.^17^ In fact, low-temperature-processed C-CEs (1) can be easily integrated with established effective CTLs. Meanwhile, they are (2) compatible with large perovskite crystals, which are not constrained by the pore size of mesoporous C-CEs in C-PSCs; (3) applicable to flexible configurations, enabling low-cost, roll-to-roll manufacturing processes; and (4) highly scalable since their short sintering optimally matches with the highest production rate reported so far for the underlying PSC structures (in the order of 0.1 m^2^ min^–1^).^48,49^

**Figure 1:**
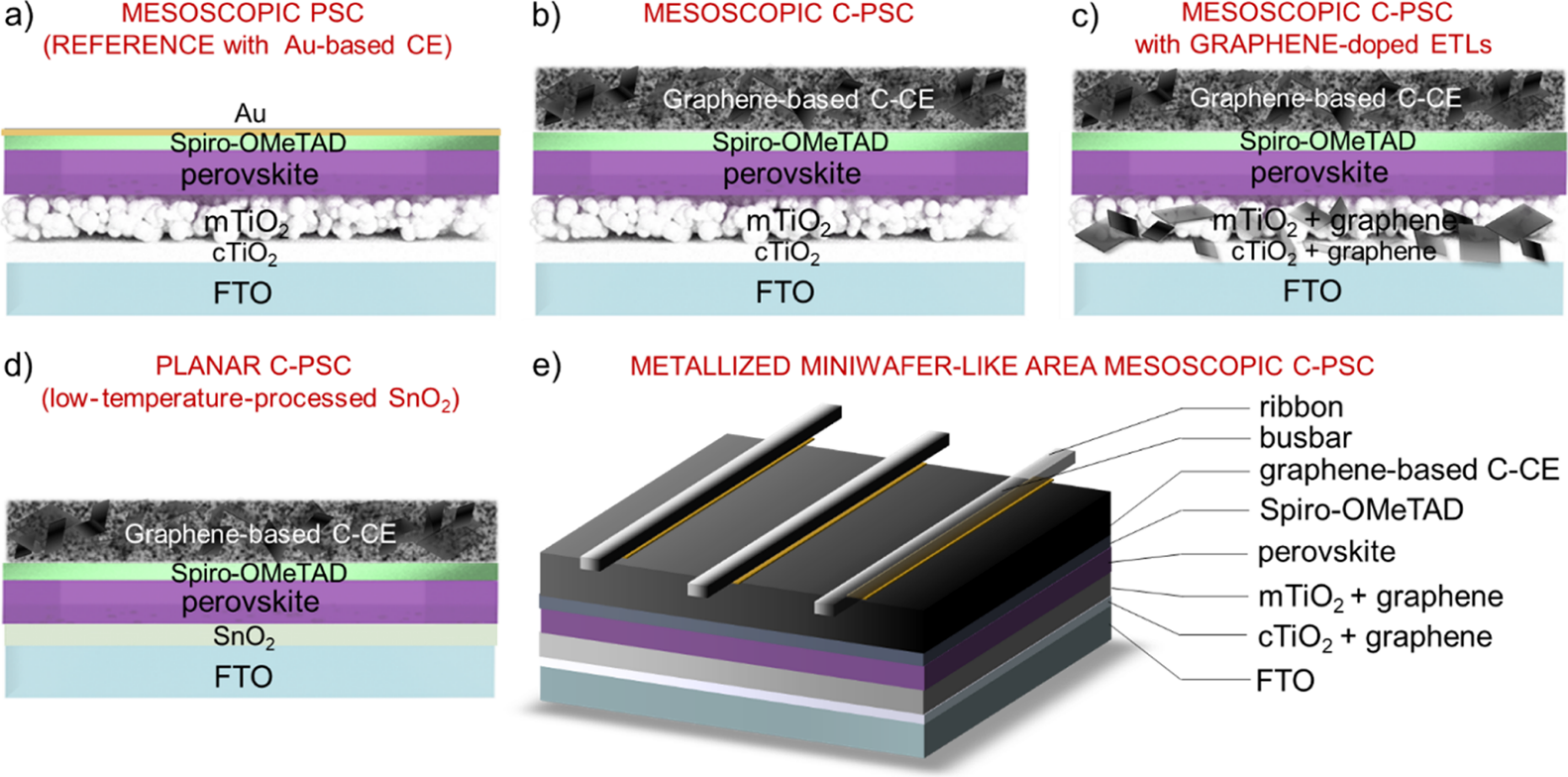
Schematics of the investigated n–i–p device architectures: (a) mesoscopic PSCs (reference cells using Au-based CE); (b) mesoscopic C-PSCs based on graphene-based C-CE; (c) mesoscopic C-PSCs with graphene-doped ETLs (cTiO_2_ + graphene and mTiO_2_ + graphene); (d) planar C-PSCs based on low-temperature-processed SnO_2_ ETL; and (e) metallized miniwafer-like area mesoscopic C-PSC, conceived as a replacement of the serial PSM configuration for the fabrication of reliable perovskite solar panels.

By rationalizing the technological status of C-PSCs, we report an affordable and facile fabrication of paintable C-PSCs based on C-CEs directly printed atop efficient prototypical PSC configurations (Figure 1). Small-area 0.09 cm^2^ and large-area 1 cm^2^ Au-based reference devices and paintable C-PSCs were fabricated either in mesoscopic or low-temperature-processed planar configurations (Figure 1a–d). For the mesoporous devices, graphene-doped TiO_2_ ETLs were used to improve the performance of mesoscopic C-PSCs based on pristine ETLs, in agreement with our previous works.^50−52^ The C-CEs were realized by depositing commercially available pastes based on thermoplastic binders and a high vapor pressure alcoholic solvent [i.e., isopropyl alcohol (IPA)], which does not damage the underlying layers. Despite its simplicity, our approach enables small-area (0.09 cm^2^) mesoscopic C-PSCs to achieve a maximum PCE of 15.81%. To prove their versatility and scalability, the proposed C-CEs were applied to large-area (1 cm^2^) mesoscopic devices and low-temperature-processed planar n–i–p devices based on SnO_2_ ETL, reaching PCEs of 13.85 and 14.06%, respectively. Remarkably, the PCEs of our all-printed large-area C-PSC are superior to the record-high values reported in the literature for large-area C-PSCs^20,39,42,53−58^ in the absence of back-contact metallization^39,53,59^ and additional stacked conductive components (e.g., graphite and ITO).^39,60^

Based on these results, we realized a proof-of-concept metallized miniwafer-like area C-PSC (substrate area = 6.76 cm^2^, aperture area = 4.00 cm^2^) (Figure 1e), reaching a PCE on active area of 13.86% and a geometric fill factor (FF_geom_, also called aperture ratio) of ∼87.3%, corresponding to a record-high PCE on aperture area of 12.1%. In this regard, we discuss that monolithic wafer-like area PSCs can represent all solution-processed configurations, which are more reliable than prototypical serial PSM configurations in developing practical perovskite solar plants since (1) they mitigate hysteresis-induced performance loss^61^ and hot-spot-induced irreversible material (perovskite and CTL) damage caused by reverse biases^62^ and (2) they have the potential to maximize the FF_geom_ using high-resolution metallization processes widely established in PV technologies besides the PSCs.

## Results and Discussion

### Carbon-Based Counter Electrodes

**Figure 2:**
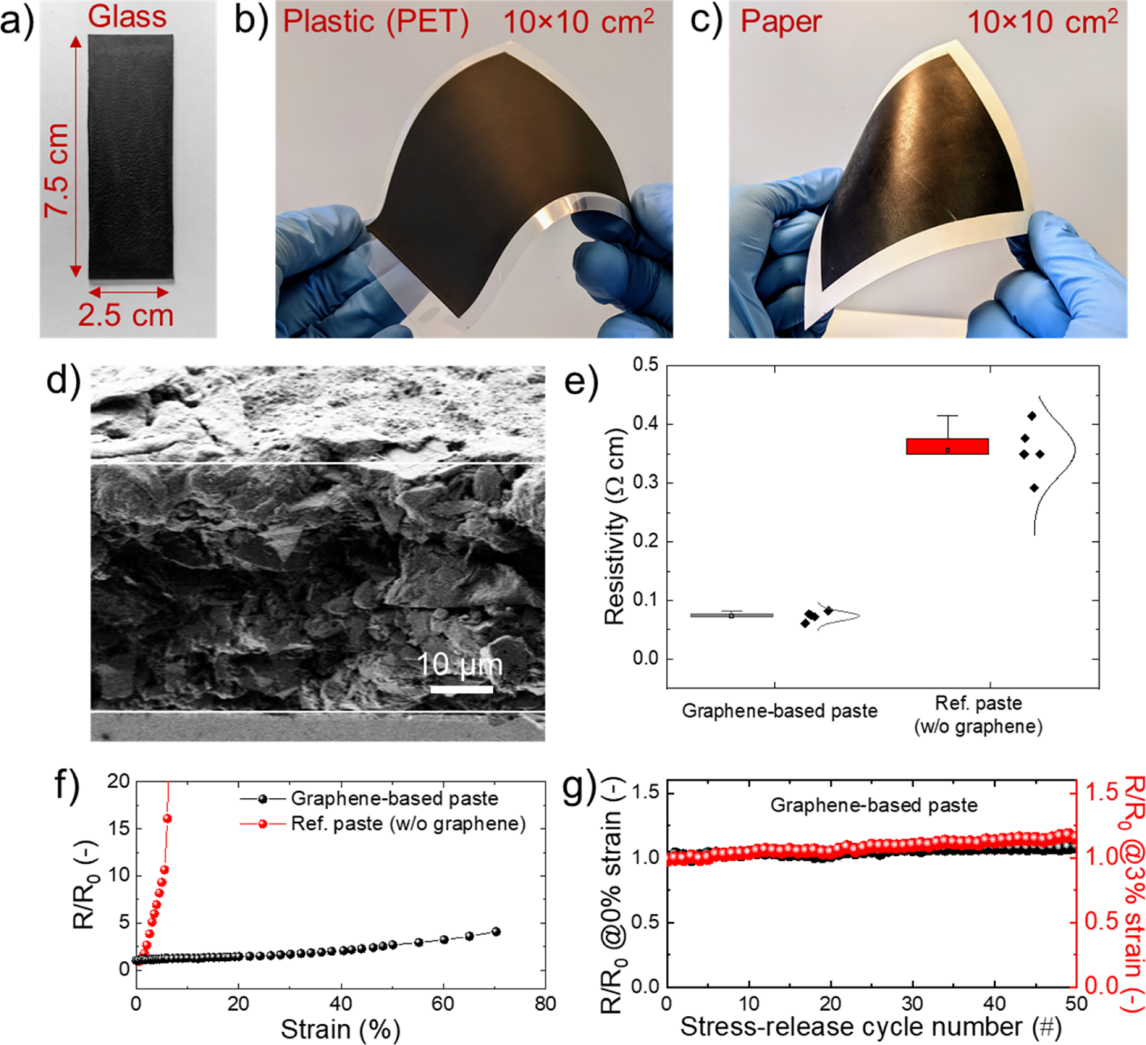
Photographs of graphene-based carbon paste deposited onto different substrates: (a) glass; (b) PET; and (c) paper. (d) Cross-sectional SEM image of a film obtained by depositing the graphene-based carbon paste (thickness *ca*. 40 μm). (e) Statistics for the electrical resistivity of the films obtained by depositing graphene-based pastes and reference pastes (w/o graphene). (f) Resistance (*R*) of the films obtained by depositing graphene-based pastes and reference pastes (w/o graphene) normalized on the initial value (strain = 0%) (*R*_0_) as a function of the strain and (g) the number of S–R cycles at a tensile strain of 3%.

The C-CEs investigated here were produced by printing commercial low-temperature-processed carbon pastes supplied by BeDimensional S.p.A.^63^ The pastes are given by a mixture of the following composition: (1) carbon black and single/few layer graphene flakes as the electrically conductive component; (2) thermoplastic polymers as the binder; and (3) high-vapor pressure alcohols, including IPA, as the solvent. The graphene flakes are produced through the evaporation and freeze-drying of a dispersion of flakes produced through scalable wet-jet milling (WJM) exfoliation of graphite, as disclosed in patent nr. WO2017/089987A1.^64^ Briefly, the WJM exfoliation process exploits a high pressure (between 180 and 250 MPa) to force the passage of the solvent/graphite mixture through perforated disks, with adjustable hole diameters (0.3–0.1 mm, named nozzle), generating shear forces that exfoliate the graphite.^65,66^ This exfoliation method provides a production rate of graphene flakes of ∼0.4 g min^–1^ (on a single WJM apparatus) and an exfoliation yield of 100%.^65,66^ These features make the WJM-produced graphene flakes affordable for massive applications (contrary to other highly conductive graphitic materials, e.g., carbon nanotubes).^67^ Differently from other chemical exfoliation and chemical/thermal reduction methods used for the production of graphene derivatives (e.g., reduced graphene oxides),^68,69^ the WJM exfoliation process preserves the chemical purity of the starting graphite. Meanwhile, it avoids the formation of functional groups and structural defects in the graphene basal planes,^65,66^ which deteriorate the ideal graphene properties, including electrical conductivity.^70,71^ By adjusting the concentration of their solid content, the pastes can be deposited on both rigid (e.g., glass) and flexible (e.g., polyethylene terephthalate, PET, and paper) substrates using common printing techniques, including doctor-blading and spray coating (Figure 2a–c) as well as spin coating (as shown hereafter). For all the cases, the deposition temperature of the paste can be inferior to 100 °C and even equal to ambient temperature. Depending on the temperature, the curing time for tens of micrometers thick films ranges from seconds/tens of seconds (for temperature around 100 °C) to tens of minutes (for ambient temperature). Figure 2d shows a cross-sectional scanning electron microscopy (SEM) image of a ∼40 μm thick film obtained by depositing the graphene-based carbon paste by doctor blading (films cured at 40 °C for *ca*. 5 min). The presence of graphene flakes is distinguishable within the binder matrix. As shown in Figure 2e, the use of graphene flakes enables the C-CEs to reach a resistivity as low as 0.07 ± 0.01 Ω cm, corresponding to an *R*_sheet_ of ∼17 Ω sq^–1^ for a thickness of 40 μm. Such an *R*_sheet_ is similar or inferior to the values reported for common transparent conductive oxide (TCO)-based CEs and C-CEs.^72,73^ Remarkably, a reference paste without graphene shows a 5-fold increase of the resistivity compared to the graphene-based paste (resistivity = 0.35 ÷ 0.06 Ω cm). Thus, graphene-based C-CEs are expected to alleviate the drawback (e.g., low FF) related to the high series resistance of current collectors in large-area solar cells.^74,75^ In addition, graphene-based films show optimal mechanical properties, which may be beneficial to tolerate thermomechanical stresses associated with natural weathering (day/night cycles, as simulated by the IEC 61215:2016 damp heat test^76^)^77^ and metallization–encapsulation processes.^78^ As shown in Figure 2f, they withstand tensile strains before neat fracture >70% (fracture strain of PET), while a reference film without graphene breaks to a tensile strain lower than 10%. Moreover, the graphene-based films optimally retain their electrical conductivity over 50 stretch–release (S–R) cycles at a tensile strain of 3%, while the films without graphene did not withstand the first release after stretching (Figure 2g).

### Device Fabrication and Characterization

To assess the effectiveness of our (graphene-based) C-CEs, small-area (0.09 cm^2^) and large-area (1 cm^2^) Au-based reference devices and paintable C-PSCs were fabricated either in mesoscopic or low-temperature-processed planar configurations (Figure 1a–d). First, prototypical mesoscopic n–i–p devices were produced with the following structure: glass/fluorine tin oxide (FTO)/cTiO_2_/mTiO_2_/perovskite/spiro-OMeTAD/C-CE (or Au-based CE) (Figure 1a,b), in which the perovskite is the triple cation Cs_0.05_(FA_0.85_MA_0.15_)_0.95_Pb(I_0.85_Br_0.15_)_3_.^52,79^ Although spiro-OMeTAD is an expensive HTL with stability issues, it was used in this work to provide proof-of-concept C-PSCs that can be prospectively modified using the most advanced, stable, and viable HTLs established in recent literature studies.^80,81^ In a second device version, both cTiO_2_ and mTiO_2_ were doped with graphene flakes (ETLs named cTiO_2_ + graphene and mTiO_2_ + graphene, respectively) (Figure 1c). To dope cTiO_2_ and mTiO_2_, the cTiO_2_ precursor solution and the mTiO_2_ paste were mixed with the commercial WJM-produced graphene dispersion in ethanol:water (see additional details in the Supporting Information). We have previously shown that graphene doping improves the electrical conductivity of cTiO_2_/mTiO_2_, thus accelerating the electron extraction toward the front CE.^50−52^ Furthermore, graphene flakes regulate the perovskite crystal growth over mesoscopic scaffolds, including m-TiO_2_, increasing the reproducibility of the active layer deposition.^82^ Meanwhile, graphene flakes act as stabilizers for the perovskite, slowing down charge thermalization processes, potentially enabling advanced hot-carrier extraction- and collection-exploiting device concepts.^83^ The photographs of representative small-area and large-area mesoscopic devices using graphene-doped ETLs are reported in Figure S1. Apart from TiO_2_-based ETLs, SnO_2_-based ETLs were also produced through spin coating for large-area 1 cm^2^ low-temperature-processed PSCs (Figure 1d). The graphene-based C-CEs were deposited by spin coating the as-supplied graphene-based paste directly onto spiro-OMeTAD. As shown in our previous works on PSCs and PSMs, by depositing two-dimensional MoS_2_ inks as buffer layers between spiro-OMeTAD and Au CEs,^52,84,85^ alcoholic solvents can preserve the integrity of the underlying perovskite/spiro-OMeTAD structure. The protocols used for the material preparation and device manufacturing follow those reported in previous literature^86^ and are described in detail in the Supporting Information. In addition to 1 cm^2^ area PSCs, metallized miniwafer-like area mesoscopic C-PSCs were produced on 6.76 cm^2^ substrates (see additional details of the layout in the Supporting Information). Both front electrode (FTO) and back C-CE were metallized by three Au stripes, as depicted in Figure 1e, to avoid series resistance losses. Such a prototype architecture aims to mimic the ones used for wafer-area solar cells,^87^ including the massively commercialized Si-based SCs.^88^ Hereafter, the advantages of monolithic wafer-like area PSCs compared with prototypical (mini)module configurations (i.e., serially connected solar cells reaching a total active area approaching to the wafer scale^40,41,52^) will be thoroughly discussed.

**Figure 3:**
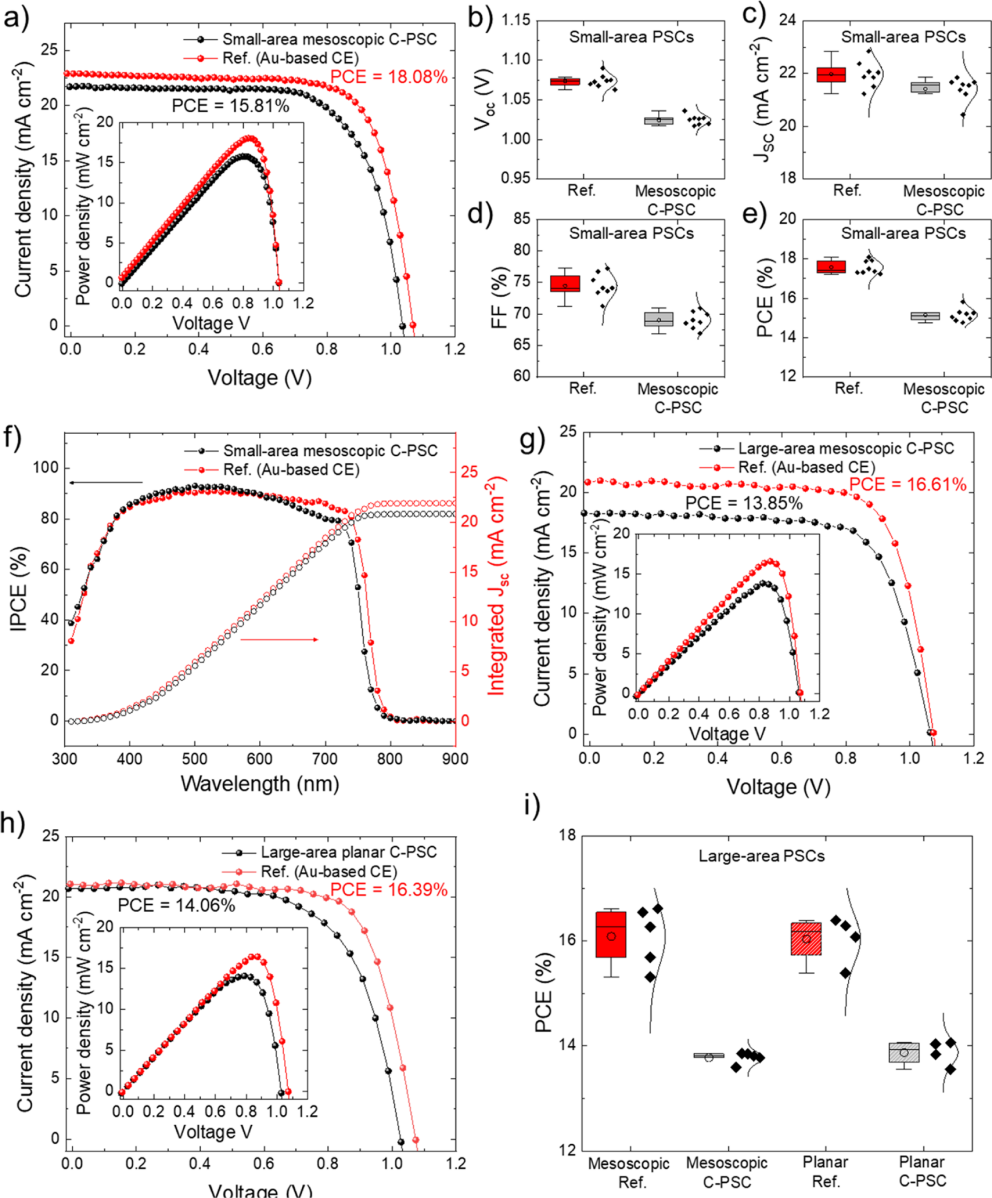
(a) *J*–*V* curves (reverse voltage scan) measured for the most efficient small-area (active area = 0.09 cm^2^) mesoscopic C-PSC using graphene-doped ETLs and Au-based reference. The inset panel shows power density vs voltage plots measured for the same devices; (b–d) statistics of the PV figures of merit measured in the reverse voltage scan mode for the small-area (active area = 0.09 cm^2^) mesoscopic C-PSCs using graphene-doped ETLs and Au-based references: *V*_oc_ (b), *J*_sc_ (c), FF (d), and PCE (e). (f) IPCE spectra for representative small-area (active area = 0.09 cm^2^) mesoscopic C-PSC using graphene-doped ETLs and Au-based reference. (g) *J*–*V* curves (reverse voltage scan) measured for the most efficient large-area (active area = 1 cm^2^) mesoscopic C-PSC using graphene-doped ETLs and Au-based reference. The inset panel shows power density vs voltage plots measured for the same devices. (h) *J*–*V* curves (reverse voltage scan) measured for the most efficient large-area (active area = 1 cm^2^) low-temperature-processed planar C-PSC and Au-based reference. The inset panel shows power density vs voltage plots measured for the same devices. (i) Statistics of the PCE data measured in the reverse scan mode for the large-area (1 cm^2^) mesoscopic and planar C-PSCs and Au-based references.

Figure 3a reports the current–voltage (*J*–*V*) curve (reverse voltage scan) measured for the most efficient small-area (active area = 0.09 cm^2^) mesoscopic C-PSC using graphene-doped ETLs under 1 sun illumination in comparison with the *J*–*V* curves measured for the best reference cell using Au-based CEs. The PCE of the mesoscopic C-PSCs is as high as 15.81%, representing a 12.5% decrease relatively to the Au-based reference (PCE = 18.08%). Remarkably, our C-PSCs exhibit a high *V*_oc_ of 1.04 V and an FF of 70.44%, which is not easily reported for paintable C-PSCs in the absence of back-contact metallization^39,53,59^ or additional stacked conductive components (e.g., graphite and ITO).^39,60^ The inset of Figure 3a shows the power density versus voltage plots of the champion devices, indicating maximum power densities of 15.86 and 18.10 mW cm^–2^ for mesoscopic C-PSCs and Au-based reference, respectively. Figure 3b–e shows the statistics of the PV figures of merit [namely, open-circuit voltage (*V*_oc_), short-circuit current density (*J*_sc_), FF, and PCE] for the investigated small-area mesoscopic PSCs, as extracted by their *J*–*V* curves in the reverse voltage scan mode. Clearly, the mesoscopic C-PSCs show reproducible results over various cells, resulting in an average PCE of 15.15%. The statistics of the PV figures of merit extracted from *J*–*V* curves acquired in the forward voltage scan mode are reported in Figure S2, indicating a similar hysteresis behavior for the C-PSCs and Au-based references (average PCEs of 16.02 ± 0.56% and 13.77 ± 0.31%, respectively). The characterization of the C-PSCs obtained with undoped ETLs is reported in the Supporting Information (Figure S3). The average PCE of the device with undoped ETLs decreases by ∼8.6% compared to devices with graphene-doped ETLs, confirming the beneficial role of the graphene doping for the ETLs in mesoscopic devices.^50,52,82,89^Figure 3f shows the incident photon-to-current efficiency (IPCE) spectra measured for representative small-area mesoscopic C-PSCs and the Au-based reference, with PCEs of 15.81 (champion device) and 17.36%, respectively. The integrated *J*_sc_ values match the ones extrapolated from the *J*–*V* curves measured for the most efficient devices. The IPCE data reveal that major IPCE losses in the C-PSCs compared to the Au-based reference occur in the spectral region between 630 and 800 nm. Even though we do not have a clear explanation for the IPCE loss in our C-PSCs, we speculate that the deposition of the carbon paste may affect the original devices’ interfaces, causing a recombination of the charges photogenerated by the light with the lowest energies. Although the solvent used for the graphene-based carbon paste, namely, IPA, has been proved to be compatible with the perovskite in our previous works,^52,84^ it may still have some influence on the quality of the spiro-OMeTAD HTL and related interfaces. In this context, the combination of our graphene-based carbon paste technology with advanced HTLs alternative to spiro-OMeTAD is promising to further boost the PV performances obtained in our work while decreasing the overall device costs. Figure 3g reports the *J*–*V* curves (reverse voltage scan) measured for the champion large-area (active area = 1 cm^2^) mesoscopic C-PSCs (C-CE produced by spin coating the graphene-based carbon paste) and the corresponding Au-based reference. The cells achieved PCEs of 13.85 and 16.61%, respectively. In addition, mesoscopic C-PSCs were also produced by depositing the graphene-based C-CEs through the doctor blading method, reaching a maximum PCE as high as 12.33% (Figure S4, Table S1). Similar C-PSC architectures with C-CEs produced by depositing a commercially available carbon paste, instead of our graphene-based carbon paste, reached a maximum PCE of 8.7% (see additional details in Supporting Information, Figure S5), which was significantly inferior to the PCEs reached by our device. The superior PV performances obtained using the graphene-based pastes compared to those achieved using other commercial carbon pastes may be related to the different solvents used in paste formulation (IPA for the graphene-based paste investigated in our work; 2-(2-ethoxyethoxy)ethyl acetate, 2-methylnaphthalene, and pentylbenzene for the commercial paste used for comparison), as well as to the optimal mechanical and electrical properties provided by the graphene flakes to the C-CEs.

In addition, HTL-free C-PSCs were also produced by eliminating the spiro-OMeTAD HTL from the device architecture. The HTL-free prototypes reached a PCE of 9.62% (Figure S6), which is promising for the realization of low-cost PSCs. The stability of the small-area cells was evaluated through ISOS-D-1 (shelf life at ambient temperature and relative humidity) and ISOS-D-2 (shelf life at 85 °C and ambient relative humidity) protocols^90^ on unencapsulated devices (Figure S7), as described in ref (91). Under the ISOS-D-1 test, the C-PSCs and Au-based references exhibited a similar stability. In particular, all the devices retained more than 90% of their initial PCE (average normalized PCE after 360 *h* > 93%). Under the ISOS-D-2 test, the Au-based references exhibited a T_80_ lifetime (here defined as the time span in which the average normalized PCE of the devices is equal to 80%) of 51.9 h, while the C-PSC reached a T_80_ of 173.1 h, indicating a stability improvement compared to the Au-based reference. Nevertheless, the stability data of the unencapsulated devices indicate that the C-CEs improve the overall stability of the devices compared to the case of Au-based CEs. In this context, the recent development of advanced encapsulants is promising for the PSC technology^13^ and such encapsulants be prospectively applied to our C-PSCs. The replacement of spiro-OMeTAD with more stable HTL may also be beneficial to further improve the stability of our C-PSCs. To further extend the applicability of our C-CEs while exploiting their full potential, large-area (1 cm^2^) low-temperature-processed planar C-PSCs were fabricated using SnO_2_ ETLs. Figure 3h shows the *J*–*V* curve (reverse voltage scan) of the champion large-area planar C-PSC and the corresponding Au-based reference. Remarkably, the planar C-PSCs reached a PCE as high as 14.06%, corresponding to an only 14.2% decrease relatively to the Au-based reference (PCE =16.36%). Figure 3i summarizes the statistics for the PCEs measured for the large mesoscopic and planar devices. The statistics for the other figures of merit for the large-area devices are reported in the Supporting Information (Figure S8).

**Figure 4:**
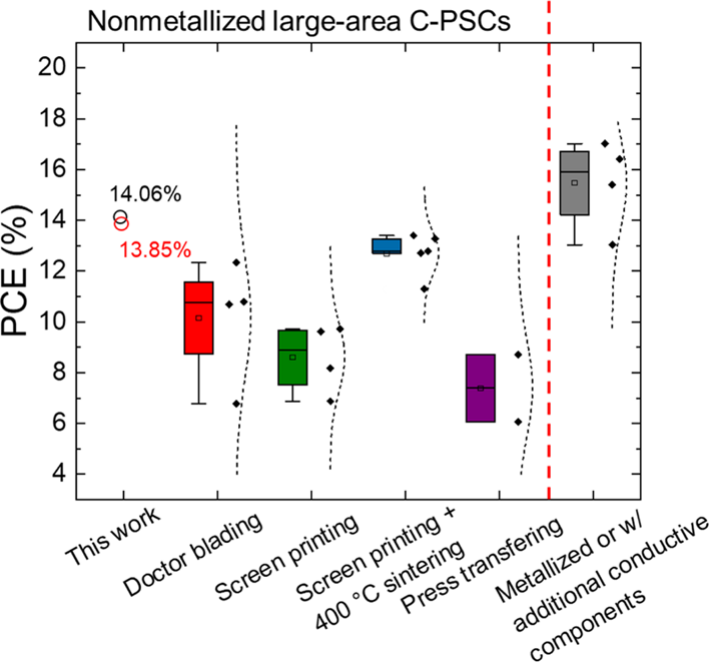
Statistics of PCEs of large-area C-PSCs reported in relevant literature studies.^20,29,39,42,53−60,92−96^ The C-PSCs are classified according to the C-CE deposition/application method. The last C-PSC class refers to C-PSCs with metallized C-CE (i.e., Cu grid^53^ or Al foil^39^) or additional conductive components stacked on C-CE (i.e., graphite paper^39^ or ITO^60^).

The PCEs achieved by our 1 cm^2^ area C-PSCs are among the highest reported values for large-area C-PSCs^20,39,42,53−58^ in the absence of back-contact metallization^39,53,59^ or additional stacked conductive components (e.g., graphite and ITO)^39,60^ or vacuum thermally evaporated protective metallic layers (e.g., Cr)^58^ (Figure 4 and Table S2). The results demonstrated here are ascribed to both the high conductivity of graphene-based C-CE (see Figure 1) and the compatibility of the low-temperature processable and alcoholic solvent-based carbon pastes with the underlying PSC layers.

**Figure 5:**
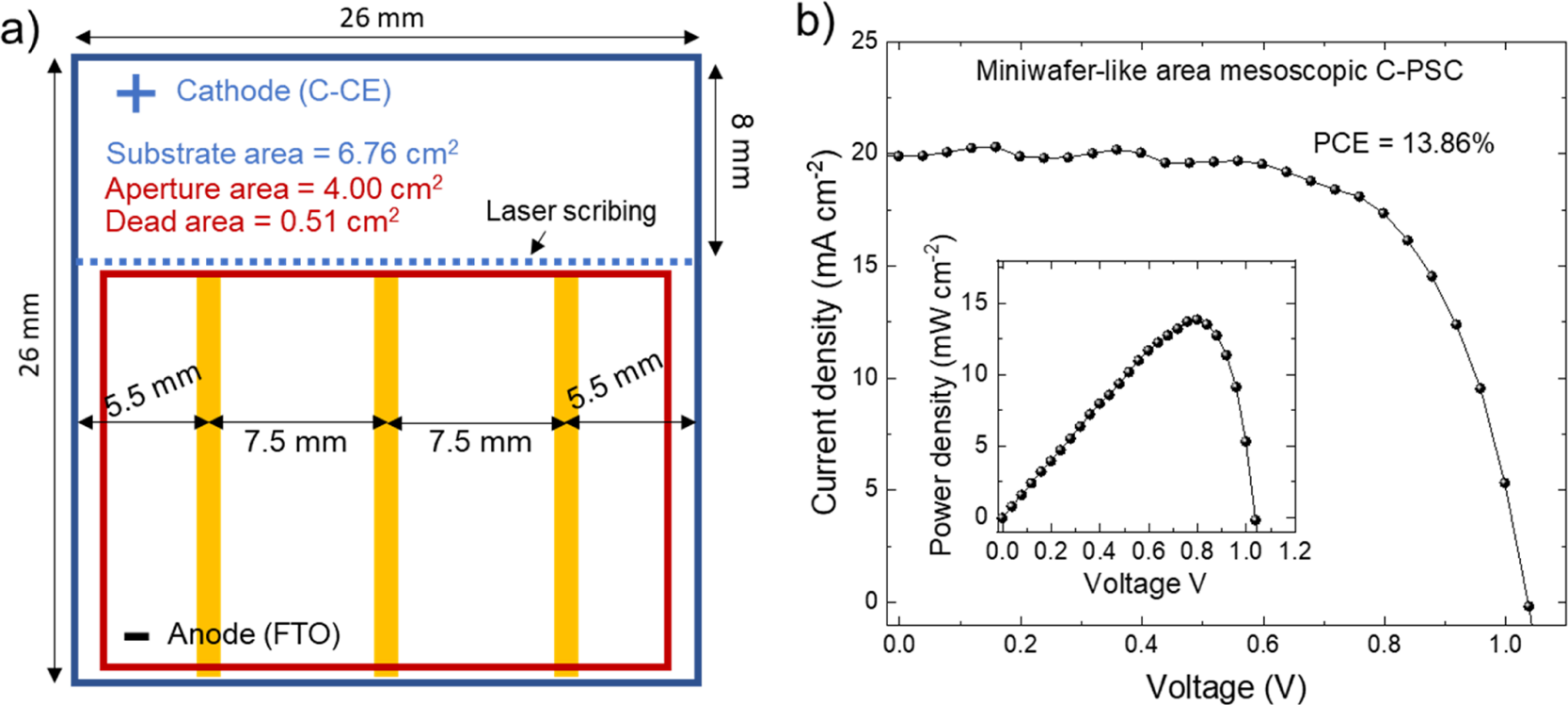
(a) Layout of the miniwafer-like area mesoscopic C-PSCs using graphene-doped ETLs, showing the front contact (i.e., FTO) Au-based metallization. A nearly specular Au-based metallization layout was used for the C-CE, as shown by the photograph of the device from its back contact (Figure S8). The aperture area corresponds to the region delimited by the red line. (c) *J*–*V* curve (reverse voltage scan) measured for the miniwafer-like area C-PSC (aperture area = 4.00 cm^2^, dead area = 0.51 cm^2^). The inset panel shows the power density vs voltage plot measured for the same device, while the right one is a photograph of the miniwafer-like area C-PSC.

After assessing the reproducible fabrication of large-area C-PSCs, metallized miniwafer-like area C-PSCs were produced on 6.76 cm^2^ substrates (aperture area = 4.00 cm^2^) according to the layout shown in Figure 5a for the front contact. Figure S9 shows the photographs of the device from its back contact (i.e., the C-CE). Both the front electrode (FTO) and back C-CE were metallized by three thermally evaporated, thin (100 nm) Au stripes (width = 1 mm) to avoid series resistance losses with upscaling of the cell area.^75,97^ In fact, electrode collection paths longer than 1 cm cause significant power loss or FF reduction due to the intrinsic resistance of the current collectors (e.g., in the order of 10 Ω sq^–1^ for prototypical TCOs, including FTO).^98^ We are aware that the accurate design of the grid architecture needs to consider several factors, for example:^97,98^ (1) the loss of the active area available for illumination; (2) the physical thickness (height) of the grid lines to achieve the lowest possible grid resistances while maintaining compatibility with the thin-film junction forming the solar cell to avoid shorting defects; and (3) the spacing and arrangement of the grid lines to reduce the carrier collection path length to less than the critical length defined by the sheet resistance of the current collectors. Despite the complexity of the grid design, it was not our goal to optimize the layout of the proposed metallized large-area device, this task being more convenient to be implemented on devices even larger than the ones investigated by this work. Nevertheless, our prototype architecture aimed to prove the possibility to use metallic grids typically applied to wafer-area solar cells (e.g., Si solar cells)^87,88^ to decrease the series resistance associated with both front and back current collectors. To accomplish this objective, we opted for a mesoscopic configuration to enter in the so-called “thick-junction regime”.^97,99^ In fact, the ETL layers (graphene-doped cTiO_2_ and graphene-doped mTiO_2_) that cover the Au-metallized FTO approach the μm scale thickness so that they avoid possible electrical shorts raised in the presence of the nonhomogeneous perovskite active layer (which is still plausible over large areas, i.e., ≫ 1 cm^2^). Since our device prototype combines solution-processed C-CE, the replacement of thermally evaporated Au stripes with solution-processed metal grids may easily enable solution-processed wafer-like area PSCs.^99^Figure 5b shows the *J*–*V* curve (reverse voltage scan) measured with our metallized miniwafer-like area mesoscopic C-PSC, which achieved a PCE of 13.86% [value calculated on the active area, i.e., (aperture area—dead area)], which is comparable to the PCE measured for the champion 1 cm^2^ area mesoscopic PSCs (PCE = 13.85%). This means that the device layout effectively avoids power losses originated by the electrical resistance of current collectors (FTO and C-CE), and the metallization is compatible with the upscaling of the device area. These results should spur research studies toward the realization of viable wafer-like area (i.e., 5/6 inches square) (C-)PSCs by means of solution-processed techniques, replacing the current (mini)module configurations based on the serial connection of perovskite subcells.^41,43,100^ The next section reports some technical considerations which justify the importance, according to our point of view, of moving from the serial PSM configurations to wafer-like area PSCs.

### Serial Perovskite Solar Minimodules or Monolithic Wafer-like Area Devices? Technical Considerations and Perspectives

Serial PSM configurations have been widely established to intrinsically avoid power losses originated by the electrical resistance of large-area transparent electrodes without requiring any additional metallization of the transparent electrodes.^41,43,100^ It is noteworthy that serial module configurations have also been established in other thin-film PV devices,^101^ where laser scribing processes can be used to achieve a high FF_geom_,^102,103^ defined as the ratio between the active area and the aperture area (active area + dead area) of the device. In fact, the serial module configuration keeps the current as small as that of each subcell while increasing the output voltage up to the sum of voltage of each subcell. However, two drawbacks arise for such architecture:^104^ (1) it is highly sensitive to the cell electrical current mismatches,^105^ which are introduced by layer inhomogeneities as well as shadows on the device area;^106^ (2) the patterning processes (e.g., laser scribing) established on Au-based CEs to realize serially connected subcells while minimizing the size of the “dead areas”^107^ cannot be directly applied to micrometers thick C-CEs.^44^ More in detail, shaded solar cells produce lower electrical current than the unshaded ones and force the in series-connected unshaded cells to deliver a low electrical current compared to their nominal ones.^105,106^ Furthermore, the shaded cells are forced to operate in reverse bias by the other cells in the string to conduct the higher electric current of the latter and eventually act as a load dissipating the power that is generated by the unshaded cells. This effect can result in the so-called “hot-spot”-induced malfunction,^108^ irreversibly damaging the entire solar module. These degrading effects are well-known for commercially available PV technologies^109^ but are rarely discussed for PSCs,^61,62,110^ in which “hot-spot” effects can be even more deleterious because of the limited thermal stability of perovskite (as well as several CTLs) at temperatures higher than 100/120 °C.^7^ In principle, bypass diodes (BPDs) may be used to bypass the irradiated solar cells.^111,112^ This approach is commonly used in commercial Si solar modules, in which a BPD is connected in parallel to strings of 15–24 cells connected in series to prevent shaded cells from reaching the junction breakdown.^112^ However, we expect two crucial complications on the use of BPDs for the case of perovskite solar minimodules. (1) The current generated in the unshaded subcells must be transported toward the PBDs parallel to the shaded cells through the current collectors (e.g., in an n–i–p configuration, TCO from the front side, and Au or C-CE from the back side), which must therefore display low electrical resistance values. Unfortunately, the TCO current collectors have resistivity in the order of 10 Ω sq^–1^, which is insufficient to collect the current toward the BPDs, causing unreliable reverse-bias protection. (2) During the activation of a BPD in the presence of one shaded cell, the maximum reverse voltage in a single shaded cell (|*V*_REV_|_max_) is determined by the number of cells in the string, their *V*_oc_, and the forward voltage (*V*_F_) of the BPD^113^
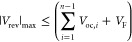
where *n* is the number of the cells in the string. Importantly, |*V*_REV_|_max_ must be inferior to the breakdown voltage (*V*_BD_) of the cell in order to avoid the junction breakdown of the cell.^113^ For the case of Si solar cells, *V*_BD_ is typically ≥12 V, *V*_oc_ is typically ≤0.75 V, and *n* = 24, safely avoiding the chance of reaching *V*_BD_ by a single cell. Unfortunately, *V*_BD_ for PSCs is typically <5 V,^61,62^ while *V*_oc_ easily reaches values above 1 V (as shown in this work). Even more, the junction breakdown in the PSCs is triggered by the accumulation of ionic defects at the interfaces, which brings irreversible degradation and hysteresis phenomena, degrading both the short- and long-term PV performance of the entire minimodule.^61^ Based on these considerations, the low V_BD_ of the PSCs would require a large amount of BPDs to prevent damages and such an amount of BPDs would inevitably increase the system costs of perovskite PVs to an uncompetitive value.^112^ In this context, wafer-like area C-PSCs could intrinsically overcome the above limits of perovskite minimodule serial configurations while providing a solution-processed cell fabrication (except for the starting TCO substrate) without recurring to expensive high-resolution film patterning processes (e.g., laser scribing),^114^ whose implementation is still unreported on tens of micrometers thick C-CEs. To the best of our knowledge, just screen printing and one example of mechanical scribing^44^ are reported for C-CEs, resulting in FF_geom_ lower than 80% (more commonly ≤70%).^44^ In our basic layout, by excluding the area left for the top and bottom contacts, the FF_geom_ is ∼87.3%, which is already comparable to the highest values reported for laser-scribed PSMs using Au as the CE^114,115^ and superior to the FF_geom_ reported for carbon PSMs.^20,40−42,44,53,55,56,116^Table S3 reports a summary of the PCE and FF_geom_ values obtained for PSMs based on C-PSCs connected in series, as reported in literature studies.^20,40−42,44,53,55,56^ Clearly, our technology, although the active and aperture areas are still significantly inferior to those reported by serial PSM configurations, has the potential to improve both PCE and FF_geom_ of the current state-of-the-art of wafer-area carbon perovskite PVs. In particular, to the best of our knowledge, our miniwafer-like area device shows a record-high PCE calculated on the aperture area of 12.10%, while previous carbon PSMs reported values lower than 9%.^20,40−42,44,53,55,56^ Last, screen printing of Ag pastes for the metallization of Si solar cells has recently demonstrated printed electrode widths of ∼20 μm.^117^ Therefore, the metallization of our basic layout through high-resolution screen printing is expected to drastically increase the FF_geom_ achieved by our proof-of-concept devices above 95%, overcoming the record-high FF_geom_ values achieved for Au-based PSMs produced through laser scribing.^114,115^ For the front TCO metallization, innovative current collector concepts based on thick metal grids embedded either in rigid or flexible substrates can preserve the planarity of the metallized front electrode,^118^ resulting in clear compatibility with the PSC technology, even in the so-called “thin-junction regime” (i.e., active layer and CTLs with sub-μm thicknesses),^97,99^ including planar (non-mesoscopic) PSC configurations.

## Conclusions

In summary, we have designed and realized efficient paintable C-PSCs obtained by depositing a commercial low-temperature-processed graphene-based carbon paste atop prototypical mesoscopic and planar n–i–p structures. Our small-area (0.09 cm^2^) mesoscopic C-PSCs achieved a maximum PCE of 15.81% while showing an improved thermal stability under the ISOS-D-2 protocol compared to the reference devices based on Au CEs. To prove the versatility and scalability of our technology, the proposed graphene-based CEs were applied to large-area (1 cm^2^) mesoscopic devices and low-temperature-processed planar n–i–p devices, reaching PCEs of 13.85 and 14.06%, respectively. To the best of our knowledge, these PCE values are among the highest reported for large-area C-PSCs in the absence of back-contact metallization or additional stacked conductive components or a thermally evaporated barrier layer between the CTL and the C-CE (strategies commonly used for the record-high efficiency of C-PSCs). Based on these results, we have realized a proof-of-concept metallized miniwafer-like area C-PSCs (substrate area = 6.76 cm^2^, aperture area = 4.00 cm^2^), reaching a PCE on active area of 13.86% and a PCE on aperture area of 12.10%. The obtained results prove the metallization compatibility with our C-PSCs. Last, we have discussed how monolithic wafer-like area PSCs can represent configurations more reliably than the prototypical serial perovskite solar (mini)modules based on the serial connection of subcells. In particular, wafer-like area C-PSCs have the potential to intrinsically mitigate the reverse-bias effects that cause hysteresis-induced performance loss and hot-spot-induced irreversible material damage in serial perovskite solar (mini)module configurations. Meanwhile, their compatibility with metallization processes is promising for the realization of all solution-processed wafer-like area C-PSCs with a high FF_geom_ (>90%), without recurring to expensive and poorly reproducible laser scribing processes.

## Supporting Information Available

Methods; supplementary PV characterization of small-area PSCs; photographs of small-area and large-area Au-based PSCs and C-PSCs; ISOS-D-1 and ISOS-D-2 stability tests on small-area PSCs; statistics for the PV figure of merit for large-area graphene-engineered mesoscopic and planar C-PSCs and Au-based references; and comparison between our device performances and those reported in the literature (PDF)
