# Supplementary material for: Low-Temperature Graphene-Based Paste for Large-Area Carbon Perovskite Solar Cells
Source: ACS Appl Mater Interfaces. 2021 May 10;13(19):22368–80. doi: 10.1021/acsami.1c02626 (PMC8289184; doi:10.1021/acsami.1c02626)
Supplement: Supplementary file 1 — am1c02626_si_001.pdf [file am1c02626_si_001.pdf]

SUPPORTING INFORMATION

Low-Temperature Graphene-Based Paste for Large Area Carbon Perovskite Solar Cells

Paolo Mariani,[1] Leyla Najafi,[2] Gabriele Bianca,[3,4] Marilena Isabella Zappia,[2,5] Luca Gabatel,[2] Antonio Agresti,[1] Sara Pescetelli,[1] Aldo Di Carlo,[1,6] Sebastiano Bellani,[2*] and Francesco Bonaccorso,[2,3*]*

[1] CHOSE – Centre for Hybird and Organic Solar Energy, University of Rome Tor Vergata, via del Politecnico 1, 00133 Rome, Italy

[2] BeDimensional S.p.A., Via Lungotorrente Secca 3D, 16163 Genova, Italy

[3] Graphene Labs, Istituto Italiano di Tecnologia, via Morego 30, 16163, Genova, Italy

[4] Dipartimento di Chimica e Chimica Industriale, Università degli Studi di Genova, via Dodecaneso 31, 16146 Genoa, Italy

[5] Department of Physics, University of Calabria, via P. Bucci cubo 31/C, 87036 Rende, Cosenza, Italy

[6] ISM-CNR, Istituto di Struttura della Materia, Consiglio Nazionale delle Ricerche, via del Fosso del Cavaliere 100, 00133 Roma (Italy)

Corresponding authors:

Aldo Di Carlo, email adress: aldo.dicarlo@uniroma2.it

Sebastiano Bellani, email address: s.bellani@bedimensional.it

Francesco Bonaccorso, email adress: f.bonaccorso@bedimensional.it

Methods

Materials. Mesoporous transparent titania paste (30 NR-D), formamidinium iodide (FAI) and methylammonium bromide (MABr) were purchased from GreatCell Solar. Lead(II) iodide (PbI_2), lead(II) bromide ($PbBr_2$) were purchased from TCI and cesium iodide (CsI) from GmbH. All other materials, including titanium(IV) isopropoxide (TTIP), diisopropoxytitanium bis(acetylacetonate) ($Ti(AcAc)_2$), acetyl acetone (AcAc), ethanol (EtOH) (≥ 99.8 %), acetone (≥ 99.5%), 2-propanol (IPA) (≥ 99.5%), dimethylformamide (DMF) ≥99%), dimethyl sulfoxide (DMSO) (>99%), chlorobenzene (CB) (99.8%), N-methyl-2-pyrrolidone (NMP) (> 97%), tris(2-(1H-pyrazol-1-yl)-4-*tert*-butylpyridine)cobalt(III) tri[bis(trifluoromethane)sulfonimide] (FK209) bis(trifluoromethane)sulfonimide lithium salt (LiTFSI) and 4-*tert*-butylpyridine (tBP) and graphite flakes (+100 mesh) were purchased from Sigma-Aldrich. All the materials were used as received, unless specified otherwise. Fluorine tin oxide (TO)-coated glasses (8 Ω sq^{-1}) were purchased from Pilkington. Carbon pastes with and without graphene were supplied by BeDimensional S.p.A.

The dispersion of graphene flakes in EtOH, used for the doping of the TiO_2 electron transporting layers (ETLs), was prepared through wet-jet milling (WJM) exfoliation in NMP[1,2] and exchanged into EtOH.[3,4] A four-pass protocol was used, as described in previous works.[1,5] Experimentally, a mixture including 20 L of NMP and 200 g of graphite flakes were prepared in a container and mixed by a mechanical stirrer (Eurostar digital Ika-Werke). Subsequently, the mixture was pressurized by a hydraulic piston into a processor consisting of five sets of different perforated and interconnected disks by applying a pressure in the range of 140 to 250 MPa. During this step, two jet streams originate

at the second disk, which is made of two holes with a diameter of 1 mm in diameter. Subsequently, the jet streams collide in a nozzle with a diameter of 0.3 mm between the second and the third disks. During the passage of the sample through the nozzle, the turbulence of the solvent originates the shear force causing the exfoliation of graphite.[1,2] The as-produced dispersion was cooled down by a chiller and then collected in another container. The sample was re-processed three times by three consecutive WJM machines with a nozzle of 0.2, 0.15 and 0.1 mm diameters, respectively. The as-produced graphene dispersion was subsequently ultracentrifuged at 16 000 *g* for 30 min at 15 °C using a Beckman Coulter Optima™ XE-90 centrifuge with an SW32Ti rotor. Thus, the un-exfoliated graphite was removed through the sedimentation-based separation (SBS).[6] More in detail, 80% of the supernatant was collected by pipetting after the ultracentrifugation process. The pipetted sample was dried using a rotary evaporator (Heidolph, Hei-Vap Value) at 70 °C and 5 mbar. Subsequently, 500 mL of EtOH was added to the dried sample. The sample was then dispersed using a sonic bath for 10 min. Consecutively, the sample was centrifuged at 800 g using Beckman Coulter Optima™ XE-90 centrifuge with an SW32Ti rotor. The sediments were collected discarding the supernatant. This process of decantation was repeated twice to wash out the NMP residuals. Finally, the sediments were dispersed in 200 mL of EtOH, obtaining a concentration of graphene flakes of 0.9 mg mL^{-1}.

The graphene flakes used for the formulation of the pastes were produced in form of dispersions through the WJM method in NMP, as described above. The as-obtained graphene dispersion in NMP was dried in form of powder by means of a freeze dryer machine, to obtain the final freeze-dried graphene powder. Subsequently, the component of the pastes, including WJM-produced graphene and carbon black as the electrically conductive fillers and a thermoplastic mixture, were homogeneously mixed in IPA. Graphene-free carbon pastes were produced by following the same protocol used for graphene-based carbon paste, except for using a mixture of carbon black and graphite as the electrically conductive fillers with the same weight percentage relatively to the solid content. The weight content of the electrically conductive contents was optimized to achieve to best trade-off between electrical and mechanical performances, the most relevant ones are summarized in **Figure 2** of the main text.

Devices' fabrication. The reference perovskite solar cells (PSCs) using Au as the counter electrode (CE) were fabricated by following to the protocols reported in ref. 7 for both small-area (active area = 0.09 cm^2) and large-area (active area = 1 cm^2) mesoscopic and planar n-i-p- structures, except for the use of graphene-doped compact TiO_2 ($cTiO_2$+G) and graphene-doped mesoporous TiO_2 ($mTiO_2$+G) in mesoscopic devices. The $cTiO_2$+G and $mTiO_2$+G layers were obtained by modifying the protocols reported for $cTiO_2$ and $mTiO_2$, respectively,[7] as resulting from the addition of 1 vol%

graphene dispersion in EtOH in the $cTiO_2$ precursor solution or $mTiO_2$ paste. The carbon perovskite solar cells (C-PSCs) were fabricated by following the protocols used for the Au-based reference, except for the replacement of Au-based CE with carbon counter electrodes (C-CEs). The latter were obtained by depositing through spin coating the graphene-based carbon pastes, keeping the substrates at ambient temperature. For the small-area devices, four pixels were obtained on the same substrate. Large-area C-PSCs were also produced by depositing C-CEs though doctor blading deposition of the graphene-based carbon paste or other commercially available carbon paste (DN-CP01, Dyenamo). For the metallized miniwafer-like area (substrate area = 6.76 cm^2; aperture area = 4.00 cm^2) mesoscopic C-PSCs, both front and back electrodes (*i.e.*, FTO and C-CE) were metallized by depositing through vacuum (10^{-6} bar) thermal evaporation three 1 mm-width stripes of 100 nm-thick Au electrode, following the layout shown in the main text (**Figure 5a**).

Materials' and devices' characterization. The carbon pastes were characterized by depositing ~40 μm-thick films through doctor blading on both glass and polyethylene terephthalate (PET) substrates. The thickness of the films was measured using a contact surface profilometer (XP-200, Ambios Technology). The sheet resistance (R_{sheet}) measurements were performed using a four-probe system (Jandel RM3000 Test Unit) on films deposited on glass substrates. The resistivity (Ω cm) was calculated from the film R_{sheet} (Ω sq^{-1}) and thickness (cm) according to the equation: resistivity = $R_{sheet} \times$ thickness. Scanning electron microscopy measurements were performed using a high-resolution field emission SEM (AURIGA, Zeiss) on films deposited on glass substrates. The curves of resistivity *vs.* strain and resistivity *vs.* the number of stress-release (S-R) cycles at tensile strain of 3% were acquired on films deposited on PET using a dual column tabletop universal testing system (model 3365, Instron) coupled with a source meter unit (dual channel 2612B, Keithley). Current density-voltage (J-V) characteristics of Au-based references and C-PSCs were acquired in air by using a solar simulator (ABET Sun 2000, class A) calibrated at AM1.5 and 100 mW cm^{-2} illumination with a certified reference Si Cell (RERA Solutions RR-1002). Incident power was measured with a Skye SKS 1110 sensor. The class was measured with a BLACK-Comet UV–vis spectrometer. The I-V scans were performed by using a scan rate of 100 mV s^{-1} for both small- and large-area devices (voltage step of 20 and 40 mV, respectively), including metallized miniwafer-like area C-PSCs. Incident Photon-to-current Conversion efficiency (IPCE) spectra were acquired by means of a home-made setup composed by a monochromator (Newport, mod. 74000) coupled with a Xe lamp (Oriel Apex, Newport) and a source meter (Keithley, mod. 2612). A home-made LabVIEW program controlled the spectra acquisition. The device data were analysed with OriginPro® 9.1 software. Stability tests were carried out by following the ISOS-D-1 and ISOS-D-2 protocols[8] on unencapsulated devices, as reported in ref. [9]

Photographs of small-area and large-area Au-based PSCs and C-PSCs using graphene-doped ETLs

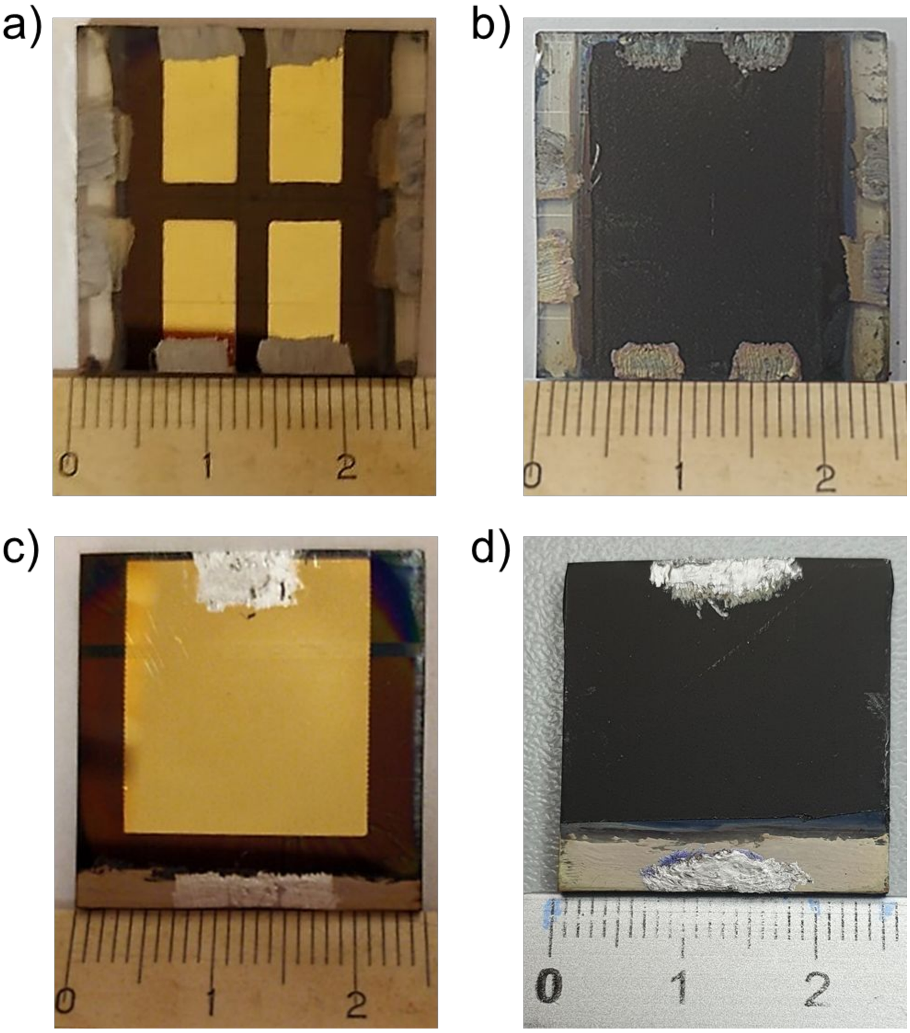

Figure S1. a.b) Photographs of representative small-area Au-based and C-CE-based mesoscopic PSCs using graphene doped ETLs (4 pixels on the same substrate), respectively. c,d) Photographs of representative large-area (1 cm^2) Au-based and C-CE-based mesoscopic PSCs using graphene-doped ETLs, respectively. All the photographs show the back-contact side of the devices.

Statistics for the photovoltaic (PV) Figures of Merit for small-area (0.09 cm^2) mesoscopic C-PSCs using graphene-doped ETLs and Au-based references measured in forward voltage scan mode

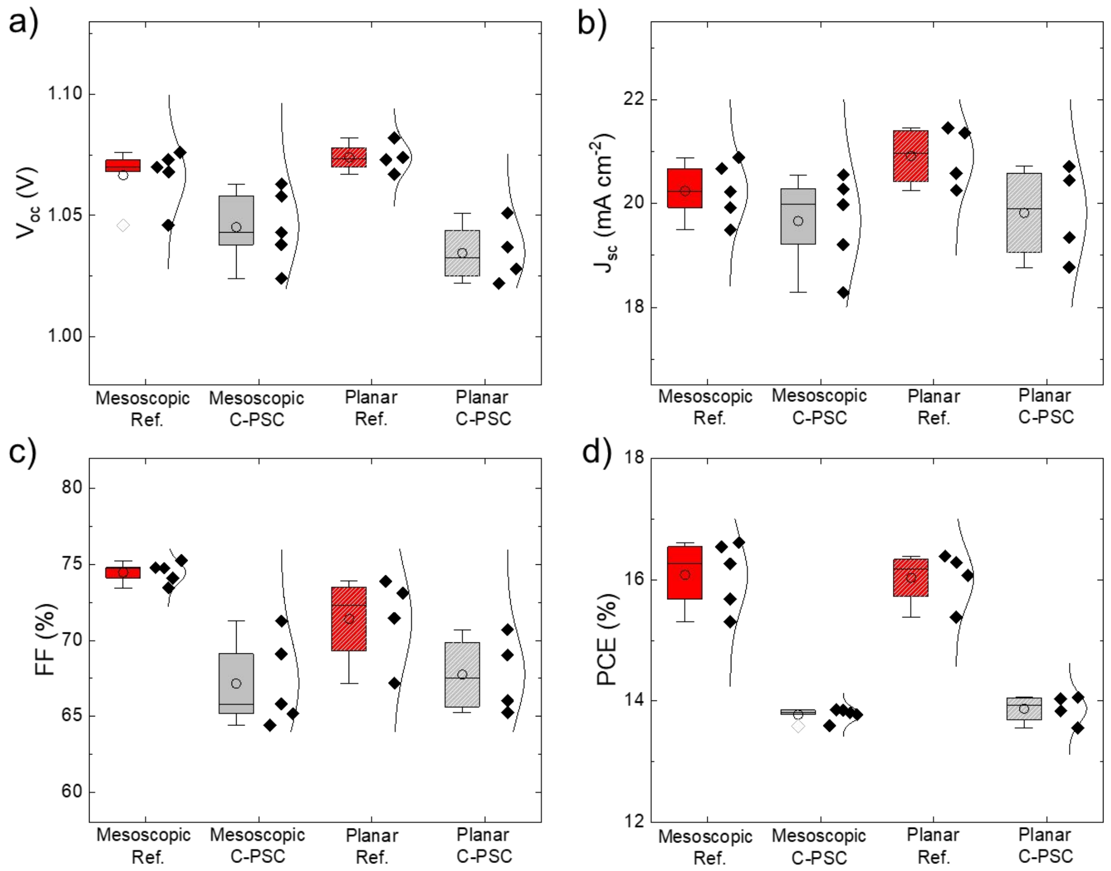

Figure S2. a-d) Statistics of the PV Figures of Merit measured in forward voltage scan mode for the small-area (active area = 0.09 cm^2) mesoscopic C-PSCs using graphene-doped ETLs and Au-based references: open circuit voltage (V_{oc}) (panel a), short circuit current density (J_{sc}) (panel b), fill factor (FF) (panel c), and power conversion efficiency (PCE) (panel d).

Characterization of small-area (0.09 cm²) mesoscopic C-PSCs with undoped ETLs and Au-based references

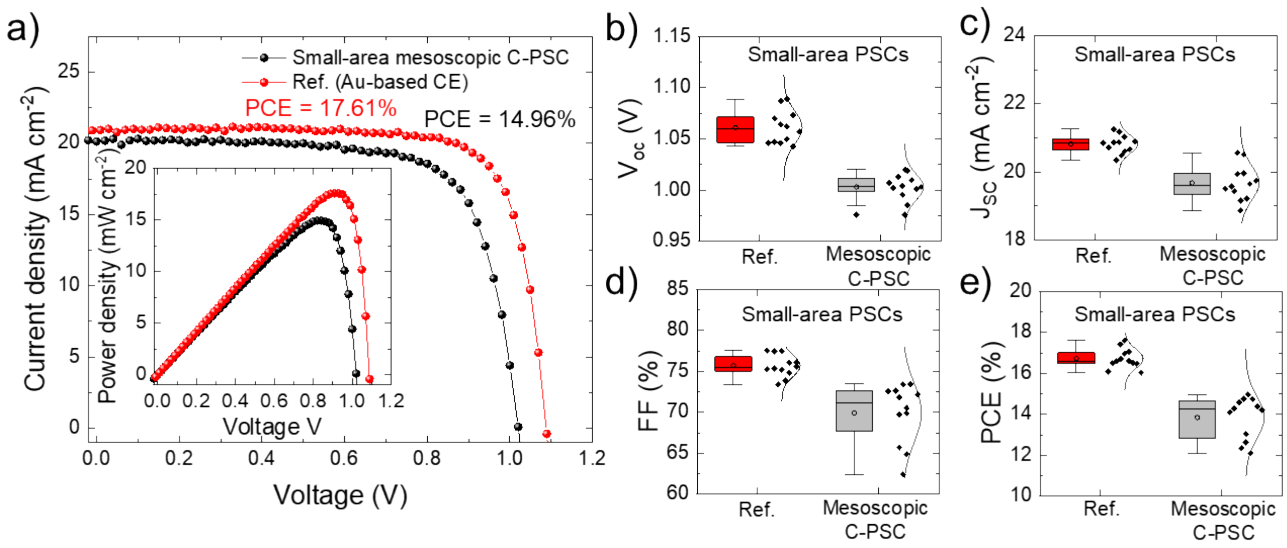

Figure S3. a) J-V curves (reverse voltage scan) measured for the most efficient small-area (active area = 0.09 cm²) mesoscopic C-PSC and Au-based reference with undoped $cTiO_2$ and undoped $mTiO_2$ ETLs. The inset panel shows the power density *vs.* voltage plots for the same devices. b-d) Statistics of the photovoltaic (PV) Figures of Merit measured in reverse voltage scan mode for the small-area (active area = 0.09 cm²) mesoscopic C-PSCs and Au-based references with undoped $cTiO_2$ and undoped $mTiO_2$ ETLs: open circuit voltage (V_{oc}) (panel b), short circuit current density (J_{sc}) (panel c), fill factor (FF) (panel d), and power conversion efficiency (PCE) (panel e).

Characterization of large-area (1 cm²) mesoscopic C-PSCs using graphene-doped ETLs and produced by depositing the graphene-based C-CEs through doctor blading method

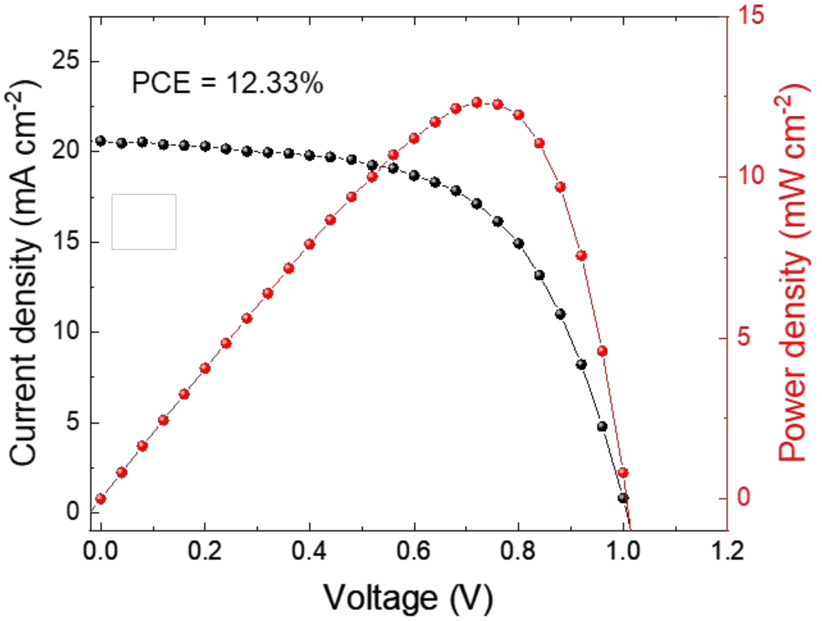

Figure S4. J-V curve (reverse voltage scan) and the corresponding power density *vs.* voltage plot measured for the most efficient large-area (active area = 1 cm²) mesoscopic C-PSC using graphene doped ETLs and produced by depositing the graphene-based C-CEs through doctor blading method.

Table S1. Photovoltaic Figures of Merit measured in reverse voltage scan mode for the mesoscopic C-PSCs using graphene-doped ETLs and produced by depositing the graphene-based C-CEs through doctor blading method.

**Sample number**	**V_{oc} (V)**	**J_{sc} (mA cm^{-2})**	**FF (%)**	**PCE (%)**
1	0.942	-19.434	65.067	11.90
2	1.023	-19.415	60.125	11.94
3	1.055	-20.714	51.132	11.17
4 (champion sample)	1.035	-20.817	57.203	12.33

Characterization of large-area (1 cm^2) mesoscopic C-PSCs using graphene-doped ETLs and C-CEs produced by depositing commercial carbon pastes through doctor blading method

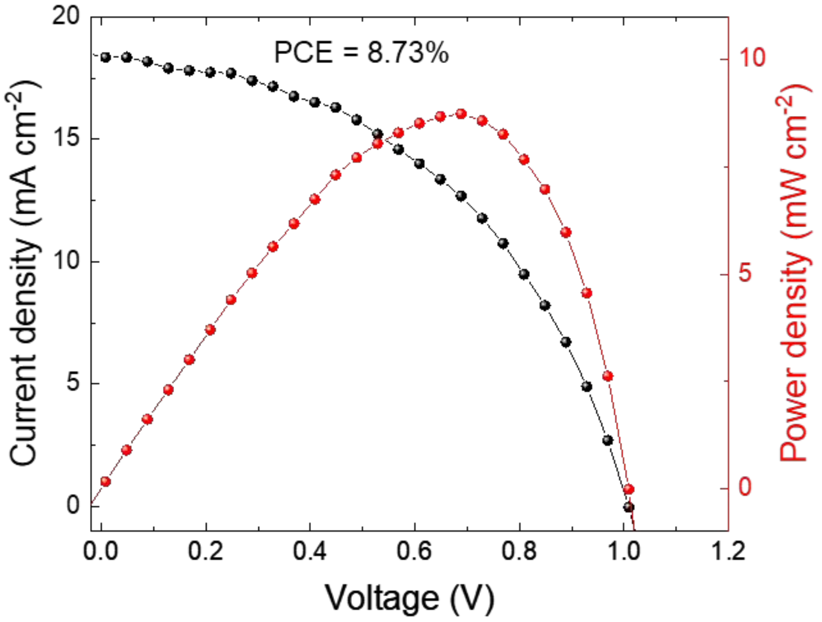

Figure S5. J-V curve (reverse voltage scan) and the corresponding power density *vs.* voltage plot measured for the most efficient large-area (active area = 1 cm^2) mesoscopic C-PSC using graphene-doped ETLs and C-CEs produced by depositing a commercially available carbon paste through doctor blading method.

Characterization of large-area (1 cm^2) mesoscopic hole transporting layer (HTL)-free C-PSCs with C-CEs produced by depositing a commercial carbon pastes through doctor blading method

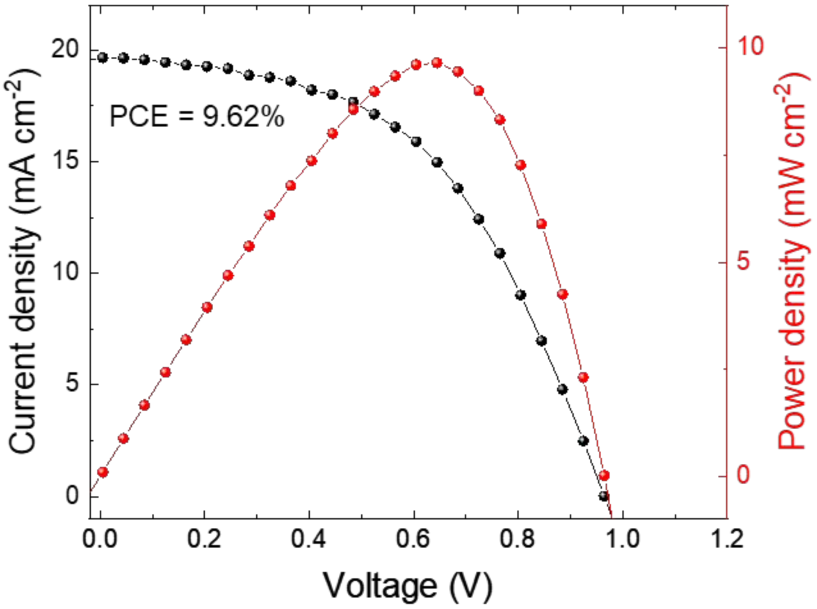

Figure S6. J-V curve (reverse voltage scan) and the corresponding power density *vs.* voltage plot measured for the most efficient large-area (active area = 1 cm^2) mesoscopic HTL-free C-PSC with C-CEs produced by depositing a commercial carbon pastes through doctor blading method.

Stability of small-area mesoscopic C-PSCs using graphene-doped ETLs

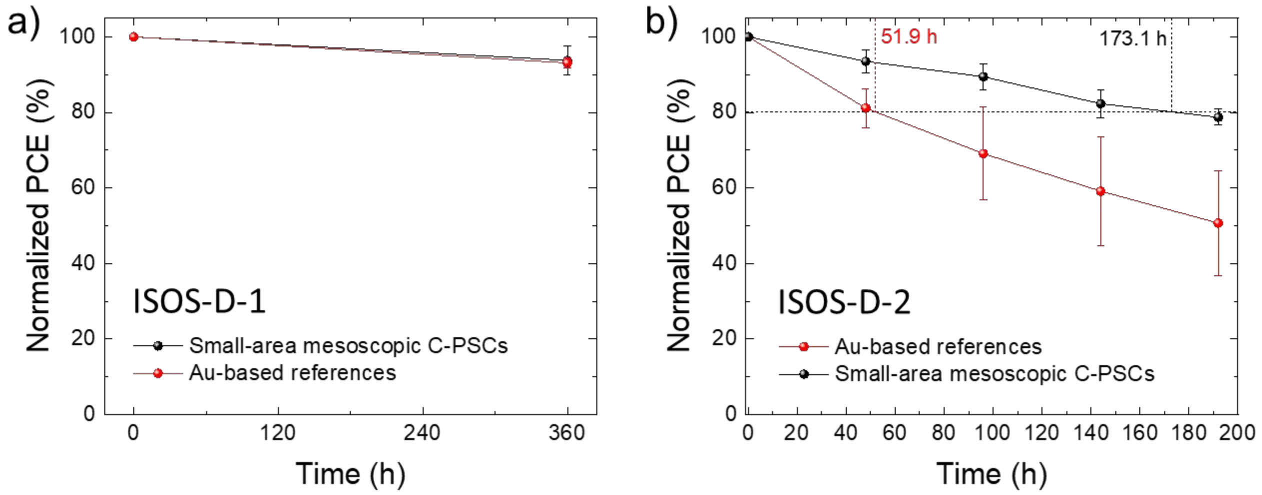

Figure S7. a,b) Stability tests of small-area mesoscopic C-PSCs using graphene-doped ETLs according to the ISOS-D-1 and ISOS-D-2 protocols, respectively. Each data point represents the average value obtained on three different devices, while the error bars represent the standard deviations for each point.

Statistics for the PV Figure of Merit for large-area (1 cm^2) mesoscopic and planar C-PSCs and Au-based references

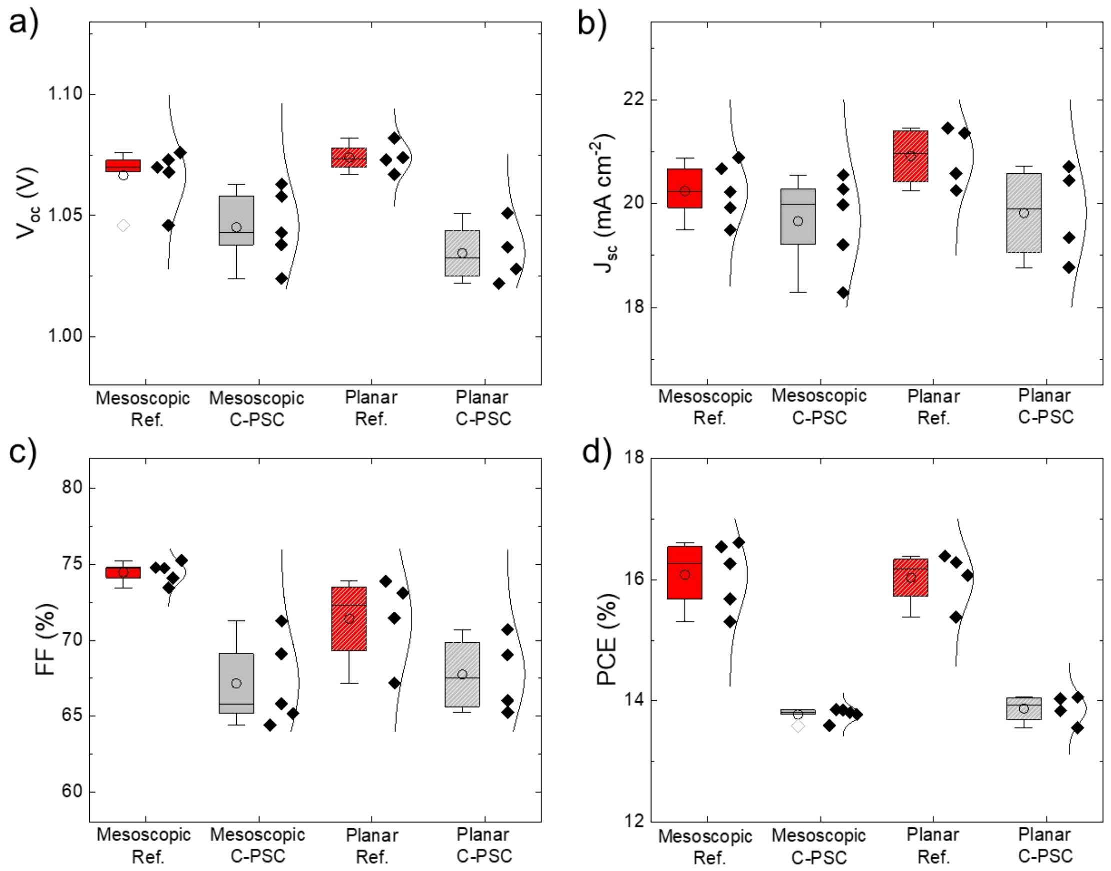

Figure S8. a-d) Statistics of the PV Figures of Merit measured for the large-area (active area = 1 cm^2) mesoscopic (based on graphene-doped ETLs) and planar C-PSCs and Au-based references: open circuit voltage (V_{oc}) (panel a), short circuit current density (J_{sc}) (panel b), fill factor (FF) (panel c), and power conversion efficiency (PCE) (panel d).

Comparison between the PCEs measured for our large-area mesoscopic and planar C-PSCs and those reported in literature for large-area C-PSCs

Table S2. Summary of the PCEs of our best large-area C-PSCs and those reported in relevant literature, with the corresponding C-CE deposition methods, cell structure and cell area.

C-CE deposition method	Cell structure	Metallization/ additional conductive component stacked on C-CE	PCE (%)	Cell area (cm^2)	Ref.
Spin coating	FTO)/TiO_2+graphene/$Cs_{0.05}(FA_{0.85}MA_{0.15})_{0.95}Pb(I_{0.85}Br_{0.15})_3$/spiro-OMeTAD/C	-	13.85	1	This work
Spin coating	FTO)/SnO_2/$Cs_{0.05}(FA_{0.85}MA_{0.15})_{0.95}Pb(I_{0.85}Br_{0.15})_3$/spiro-OMeTAD/C	-	14.06	1	This work
Doctor blading	FTO/TiO_2/$MAPbI_3$/C +PDMS	-	10.8	1	[10]
Doctor blading	(PET/)IZO/PTAA/$Cs_{0.04}(MA_{0.17}FA_{0.83})_{0.96}Pb(I_{0.83}Br_{0.17})_3$/ PCBM/C[a]	-	10.7	1	[11]
	(PET/)IZO/PTAA/$Cs_{0.04}(MA_{0.17}FA_{0.83})_{0.96}Pb(I_{0.83}Br_{0.17})_3$/ PCBM/Cr/C[a]		15.8		
Doctor blading	ITO/SnO_2/MWCNT-incorporated $FA_xMA_{1-x}PbI_yBr_{3-y}$/C	-	12.34	1	[12]
Doctor blading	Cs acetate-treated FTO/TiO_2/$MAPbI_3$/carbon	-	13.04	1	[13]
Doctor Blading	FTO/TiO_2/$CsPbIBr_2$/CuPc/C	-	6.78	1	[14]
Screen printing	FTO/ TiO_2:CsBr/$CsPbIBr_2$	-	6.88	1	[15]
Screen printing	FTO/TiO_2/$Cs_{0.06}(MA_{0.17}FA_{0.83})Pb(I_{0.84}Br_{0.16})_3$/CNT/C	-	8.18	1	[16]
Screen printing	FTO/TiO_2/$Cs_{0.05}MA_{0.16}FA_{0.79}Pb(I_{0.84}Br_{0.16})_3$/F-doped C	-	9.63	1	[17]
Printing	FTO/TiO_2/$MAPbI_3$/C	-	9.72%	1	[18]
Spray coating	FTO/SnO_2/$Cs_{0.05}MA_{0.16}FA_{0.79}Pb(I_{0.84}Br_{0.16})_3$/spiro-OMeTAD/graphene	-	< 2%	0.09	[19]
	FTO/SnO_2/$Cs_{0.05}MA_{0.16}FA_{0.79}Pb(I_{0.84}Br_{0.16})_3$/spiro-OMeTAD/graphene/ITO	ITO	16.42	1.3	
Screen printing + 400 °C sintering	FTO/TiO_2/ZrO_2/Cu-doped NiO_x/C + $(5\text{-}AVA)_x(MA)_{1-x}PbI_3$	-	12.79	0.8	[20]
Printing + 400 °C sintering	FTO/TiO_2/ZrO_2/C + $(5\text{-}AVA)_{1-x}MA_xPbI_3$)	-	12.71	0.64	[21]
Printing +400 °C sintering	FTO/TiO_2/ ZrO_2/Co_3O_4/C+ $(5\ AVA\text{-}I)_xMA_{1-x}PbI_3$	-	13.27	0.09	[22]
Screen printing + 400 °C sintering	FTO/TiO_2/ZrO_2/C + $(5\text{-}AVA)_x(MA)_{1-x}PbI_3$	-	11.3	1	[23]
		Cu grid	13.4		
Doctor blading + press transfer	FTO/SnO_2/$Cs_{0.05}(FA_{0.85}MA_{0.15})_{0.95}Pb(I_{0.85}Br_{0.15})_3$/C	-	6.06	1	[24]
	FTO/SnO_2/$Cs_{0.05}(FA_{0.85}MA_{0.15})_{0.95}Pb(I_{0.85}Br_{0.15})_3$/C/Graphite paper	Graphite paper	17.02		
	FTO/SnO_2/$Cs_{0.05}(FA_{0.85}MA_{0.15})_{0.95}Pb(I_{0.85}Br_{0.15})_3$/C/Al	Al	15.41		
Spray coating + doctor blading/press transfer	FTO/TiO_2/ $Cs_{0.04}(MA_{0.17}FA_{0.83})_{0.96}Pb(I_{0.83}Br_{0.17})_3$/C/ Al	Al	8.72	1	[25]

[a] Cr layer (5 nm) was deposited by thermal vacuum thermally evaporation

Photographs of the miniwafer-like area mesoscopic C-PSC using graphene-doped ETLs

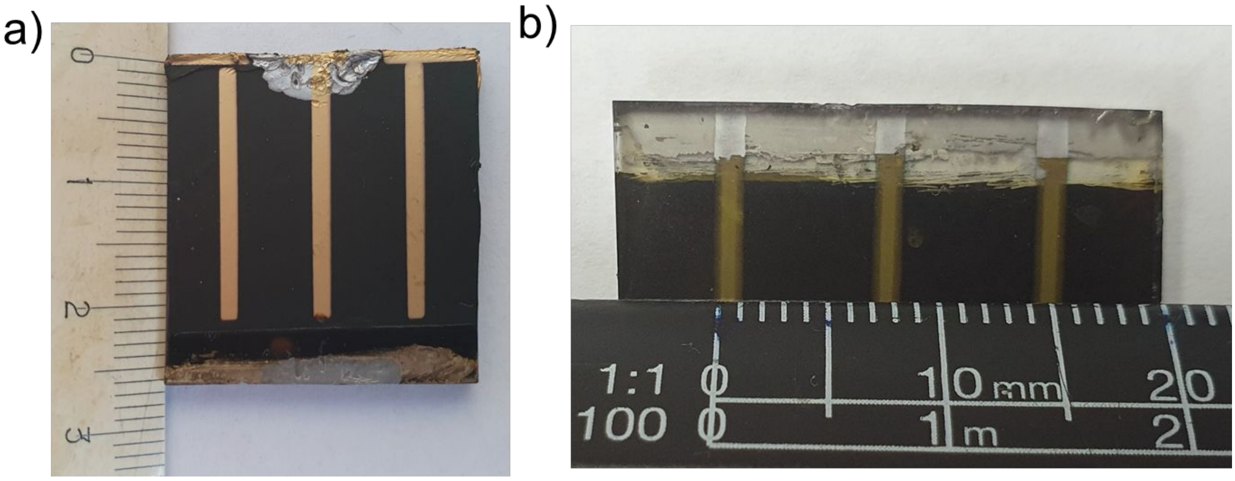

Figure S9. a,b) Photographs of representative miniwafer-like area mesoscopic C-PSCs using graphene-doped ETLs, showing their back contacts (*i.e.*, the C-CEs).

Table S3. Summary of the PCEs of our (mini)wafer-like area C-PSCs perovskite and carbon perovskite solar (mini)modules reported in relevant literature, with the corresponding C-CE deposition methods, cell structure, geometric fill factor (FF_{geom}), and cell area.

CE-deposition method	Cell structure	Active area (cm^2)	FF_{geom} (%)	PCE (%) (on active area)	FF_{geom} (%)	PCE×FF_{geom} (PCE on aperture area)	Reference
Spin coating		3.49	87.3	13.86	87.3	12.10	This work
Screen printing	$FTO/ZnO/MAPbI_3/C$	17.6	70	10.6	70	7.42	26
Printing + 400 °C sintering	$FTO/TiO_2/ZrO_2/C$ + (5-AVA)$_{1-x}$MA$_x$PbI$_3$)	47.60	47.6	10.1	47.6	4.81	21
Screen printing + 400 °C sintering	$FTO/TiO_2/ZrO_2/C$ + (5-AVA)$_x$(MA)$_{1-x}$PbI$_3$	49	49	10.4	49	5.10	27
Screen printing + 400 °C sintering	$FTO/TiO_2/ZrO_2/C$ + (5-AVA)$_x$(MA)$_{1-x}$PbI$_3$	11.7	n.d.	7.73	n.d.	n.d	23
	$FTO/TiO_2/ZrO_2/C$ + (5-AVA)$_x$(MA)$_{1-x}$PbI$_3$/Al grid			9.97			
	$FTO/TiO_2/ZrO_2/C$ + (5-AVA)$_x$(MA)$_{1-x}$PbI$_3$/Cu grid			11.05			
Printing + 400 °C sintering	$FTO/TiO_2/ZrO_2/Co_3O_4/C$+ (5 AVA-I)$_xMA_{1-x}PbI_3$	70	n.d.	11.39	n.d.	n.d.	22
Screen printing + 400 °C sintering	$FTO/TiO_2/ZrO_2/C$ + (5-AVA)$_x$(MA)$_{1-x}$PbI$_3$	128	45.5	6.6	45.5	3.00	28
Screen printing + 400 °C sintering	$FTO/TiO_2/ZrO_2/C$ + (5-AVA)$_x$(MA)$_{1-x}$PbI$_3$	17.85	71.4	9.88	71.4	7.05	29
Screen printing + 400 °C sintering	$FTO/TiO_2/ZrO_2/C$ + (5-AVA)$_x$(MA)$_{1-x}$PbI$_3$	70	70	10.74	70	7.52	30
Screen printing + 400 °C sintering	$FTO/TiO_2/ZrO_2$/Cu-doped NiO_x/C + (5-AVA)$_x$(MA)$_{1-x}$PbI$_3$	70	70	12.1	70	8.47	20

References

(1) Del Rio Castillo, A. E.; Pellegrini, V.; Ansaldo, A.; Ricciardella, F.; Sun, H.; Marasco, L.; Buha, J.; Dang, Z.; Gagliani, L.; Lago, E.; Curreli, N.; Gentiluomo, S.; Palazon, F.; Prato, M.; Oropesa-Nuñez, R.; Toth, P. S.; Mantero, E.; Crugliano, M.; Gamucci, A.; Tomadin, A.; Polini, M.; Bonaccorso, F. High-Yield Production of 2D Crystals by Wet-Jet Milling. *Mater. Horizons* **2018**, *5* (5), 890–904.

(2) E. Del Rio Castillo, A. Ansaldo, V. Pellegrini, F. Bonaccorso, Exfoliation materials by wet-jet milling techniques, P. number: W. Exfoliation Materials by Wet-Jet Milling Techniques, Patent Number: WO2017/089987A1., 2017.

(3) Lamanna, E.; Matteocci, F.; Calabrò, E.; Serenelli, L.; Salza, E.; Martini, L.; Menchini, F.; Izzi, M.; Agresti, A.; Pescetelli, S.; Bellani, S.; Del Río Castillo, A. E.; Bonaccorso, F.; Tucci, M.; Di Carlo, A. Mechanically Stacked, Two-Terminal Graphene-Based Perovskite/Silicon Tandem Solar Cell with Efficiency over 26%. *Joule* **2020**, *4* (4), 865–881.

(4) Agresti, A.; Pescetelli, S.; Palma, A. L.; Martín-García, B.; Najafi, L.; Bellani, S.; Moreels, I.; Prato, M.; Bonaccorso, F.; Di Carlo, A. Two-Dimensional Material Interface Engineering for Efficient Perovskite Large-Area Modules. *ACS Energy Lett.* **2019**, *4* (8), 1862–1871.

(5) Garakani, M. A.; Bellani, S.; Pellegrini, V.; Oropesa-Nuñez, R.; Castillo, A. E. D. R.; Abouali, S.; Najafi, L.; Martín-García, B.; Ansaldo, A.; Bondavalli, P.; Demirci, C.; Romano, V.; Mantero, E.; Marasco, L.; Prato, M.; Bracciale, G.; Bonaccorso, F. Scalable Spray-Coated Graphene-Based Electrodes for High-Power Electrochemical Double-Layer Capacitors Operating over a Wide Range of Temperature. *Energy Storage Mater.* **2021**, *34*, 1–11.

(6) Backes, C.; Abdelkader, A. M.; Alonso, C.; Andrieux-Ledier, A.; Arenal, R.; Azpeitia, J.; Balakrishnan, N.; Banszerus, L.; Barjon, J.; Bartali, R.; Bellani, S.; Berger, C.; Berger, R.; Ortega, M. M. B.; Bernard, C.; Beton, P. H.; Beyer, A.; Bianco, A.; Bøggild, P.; Bonaccorso, F.; Barin, G. B.; Botas, C.; Bueno, R. A.; Carriazo, D.; Castellanos-Gomez, A.; Christian, M.; Ciesielski, A.; Ciuk, T.; Cole, M. T.; Coleman, J.; Coletti, C.; Crema, L.; Cun, H.; Dasler, D.; De Fazio, D.; Díez, N.; Drieschner, S.; Duesberg, G. S.; Fasel, R.; Feng, X.; Fina, A.; Forti, S.; Galiotis, C.; Garberoglio, G.; García, J. M.; Garrido, J. A.; Gibertini, M.; Gölzhäuser, A.; Gómez, J.; Greber, T.; Hauke, F.; Hemmi, A.; Hernandez-Rodriguez, I.; Hirsch, A.; Hodge, S. A.; Huttel, Y.; Jepsen, P. U.; Jimenez, I.; Kaiser, U.; Kaplas, T.; Kim, H.; Kis, A.; Papagelis, K.; Kostarelos, K.; Krajewska, A.; Lee, K.; Li, C.; Lipsanen, H.; Liscio, A.; Lohe, M. R.; Loiseau, A.; Lombardi, L.; Francisca López, M.; Martin, O.; Martín, C.; Martínez, L.; Martin-Gago, J. A.; Ignacio Martínez, J.; Marzari, N.; Mayoral, Á.; McManus, J.; Melucci, M.; Méndez, J.; Merino, C.; Merino, P.; Meyer, A. P.; Miniussi, E.; Miseikis, V.; Mishra, N.; Morandi, V.; Munuera, C.; Muñoz, R.; Nolan, H.; Ortolani, L.; Ott, A. K.; Palacio, I.; Palermo, V.; Parthenios, J.; Pasternak, I.; Patane, A.; Prato, M.; Prevost, H.; Prudkovskiy, V.; Pugno, N.; Rojo, T.; Rossi, A.; Ruffieux, P.; Samorì, P.; Schué, L.; Setijadi, E.; Seyller, T.; Speranza, G.; Stampfer, C.; Stenger, I.; Strupinski, W.; Svirko, Y.; Taioli, S.; Teo, K. B. K.; Testi, M.; Tomarchio, F.; Tortello, M.; Treossi, E.; Turchanin, A.; Vazquez, E.; Villaro, E.; Whelan, P. R.; Xia, Z.; Yakimova, R.; Yang, S.; Yazdi, G. R.; Yim, C.; Yoon, D.; Zhang, X.; Zhuang, X.; Colombo, L.; Ferrari, A. C.; Garcia-Hernandez, M. Production and Processing of Graphene and Related Materials. *2D Mater.* **2020**, *7* (2), 22001.

(7) Saliba, M.; Correa-Baena, J.-P.; Wolff, C. M.; Stolterfoht, M.; Phung, N.; Albrecht, S.; Neher, D.; Abate, A. How to Make over 20% Efficient Perovskite Solar Cells in Regular (n–i–p) and Inverted (p–i–n) Architectures. *Chem. Mater.* **2018**, *30* (13), 4193–4201.

(8) Khenkin, M. V.; Katz, E. A.; Abate, A.; Bardizza, G.; Berry, J. J.; Brabec, C.; Brunetti, F.; Bulović, V.; Burlingame, Q.; Di Carlo, A.; Cheacharoen, R.; Cheng, Y.-B.; Colsmann, A.; Cros, S.; Domanski, K.; Dusza, M.; Fell, C. J.; Forrest, S. R.; Galagan, Y.; Di Girolamo, D.; Grätzel, M.; Hagfeldt, A.; von Hauff, E.; Hoppe, H.; Kettle, J.; Köbler, H.; Leite, M. S.; Liu, S.; Loo, Y.-L.; Luther, J. M.; Ma, C.-Q.; Madsen, M.; Manceau, M.; Matheron, M.; McGehee, M.; Meitzner, R.; Nazeeruddin, M. K.; Nogueira, A. F.; Odabaşı, Ç.; Osherov, A.; Park, N.-G.; Reese, M. O.; De Rossi, F.; Saliba, M.; Schubert, U. S.; Snaith, H. J.; Stranks, S. D.; Tress, W.; Troshin, P. A.; Turkovic, V.; Veenstra, S.; Visoly-Fisher, I.; Walsh, A.; Watson, T.; Xie, H.; Yıldırım, R.; Zakeeruddin, S. M.; Zhu, K.; Lira-Cantu, M. Consensus Statement for Stability Assessment and Reporting for Perovskite Photovoltaics

Based on ISOS Procedures. *Nat. Energy* **2020**, *5* (1), 35–49.

(9) Calabrò, E.; Matteocci, F.; Paci, B.; Cinà, L.; Vesce, L.; Barichello, J.; Generosi, A.; Reale, A.; Di Carlo, A. Easy Strategy to Enhance Thermal Stability of Planar PSCs by Perovskite Defect Passivation and Low-Temperature Carbon-Based Electrode. *ACS Appl. Mater. Interfaces* **2020**, *12* (29), 32536–32547.

(10) Liu, Z.; Sun, B.; Shi, T.; Tang, Z.; Liao, G. Enhanced Photovoltaic Performance and Stability of Carbon Counter Electrode Based Perovskite Solar Cells Encapsulated by PDMS. *J. Mater. Chem. A* **2016**, *4* (27), 10700–10709.

(11) Babu, V.; Fuentes Pineda, R.; Ahmad, T.; Alvarez, A. O.; Castriotta, L. A.; Di Carlo, A.; Fabregat-Santiago, F.; Wojciechowski, K. Improved Stability of Inverted and Flexible Perovskite Solar Cells with Carbon Electrode. *ACS Appl. Energy Mater.* **2020**, *3* (6), 5126–5134.

(12) Zhou, J.; Wu, J.; Li, N.; Li, X.; Zheng, Y.-Z.; Tao, X. Efficient All-Air Processed Mixed Cation Carbon-Based Perovskite Solar Cells with Ultra-High Stability. *J. Mater. Chem. A* **2019**, *7* (29), 17594–17603.

(13) Liu, T.; Wang, Z.; Lou, L.; Xiao, S.; Zheng, S.; Yang, S. Interfacial Post-Treatment for Enhancing the Performance of Printable Carbon-Based Perovskite Solar Cells. *Sol. RRL* **2020**, *4* (2), 1900278.

(14) Liu, X.; Li, J.; Liu, Z.; Tan, X.; Sun, B.; Xi, S.; Shi, T.; Tang, Z.; Liao, G. Vapor-Assisted Deposition of CsPbIBr2 Films for Highly Efficient and Stable Carbon-Based Planar Perovskite Solar Cells with Superior Voc. *Electrochim. Acta* **2020**, *330*, 135266.

(15) Zhu, W.; Zhang, Z.; Chai, W.; Chen, D.; Xi, H.; Chang, J.; Zhang, J.; Zhang, C.; Hao, Y. Benign Pinholes in CsPbIBr 2 Absorber Film Enable Efficient Carbon-Based, All-Inorganic Perovskite Solar Cells. *ACS Appl. Energy Mater.* **2019**, *2* (7), 5254–5262.

(16) Ryu, J.; Lee, K.; Yun, J.; Yu, H.; Lee, J.; Jang, J. Paintable Carbon-Based Perovskite Solar Cells with Engineered Perovskite/Carbon Interface Using Carbon Nanotubes Dripping Method. *Small* **2017**, *13* (38), 1701225.

(17) Kim, J.; Lee, G.; Lee, K.; Yu, H.; Lee, J. W.; Yoon, C.-M.; Kim, S. G.; Kim, S. K.; Jang, J. Fluorine Plasma Treatment on Carbon-Based Perovskite Solar Cells for Rapid Moisture Protection Layer Formation and Performance Enhancement. *Chem. Commun.* **2020**, *56* (4), 535–538.

(18) Chen, H.; Wei, Z.; He, H.; Zheng, X.; Wong, K. S.; Yang, S. Solvent Engineering Boosts the Efficiency of Paintable Carbon-Based Perovskite Solar Cells to Beyond 14%. *Adv. Energy Mater.* **2016**, *6* (8), 1502087.

(19) Zhang, C.; Wang, S.; Zhang, H.; Feng, Y.; Tian, W.; Yan, Y.; Bian, J.; Wang, Y.; Jin, S.; Zakeeruddin, S. M.; Grätzel, M.; Shi, Y. Efficient Stable Graphene-Based Perovskite Solar Cells with High Flexibility in Device Assembling via Modular Architecture Design. *Energy Environ. Sci.* **2019**, *12* (12), 3585–3594.

(20) Bashir, A.; Lew, J. H.; Shukla, S.; Gupta, D.; Baikie, T.; Chakraborty, S.; Patidar, R.; Bruno, A.; Mhaisalkar, S.; Akhter, Z. Cu-Doped Nickel Oxide Interface Layer with Nanoscale Thickness for Efficient and Highly Stable Printable Carbon-Based Perovskite Solar Cell. *Sol. Energy* **2019**, *182*, 225–236.

(21) Grancini, G.; Roldán-Carmona, C.; Zimmermann, I.; Mosconi, E.; Lee, X.; Martineau, D.; Narbey, S.; Oswald, F.; De Angelis, F.; Graetzel, M.; Nazeeruddin, M. K. One-Year Stable Perovskite Solar Cells by 2D/3D Interface Engineering. *Nat. Commun.* **2017**, *8* (1), 15684.

(22) Bashir, A.; Shukla, S.; Lew, J. H.; Shukla, S.; Bruno, A.; Gupta, D.; Baikie, T.; Patidar, R.; Akhter, Z.; Priyadarshi, A.; Mathews, N.; Mhaisalkar, S. G. Spinel Co_3O_4 Nanomaterials for Efficient and Stable Large Area Carbon-Based Printed Perovskite Solar Cells. *Nanoscale* **2018**, *10* (5), 2341–2350.

(23) Raptis, D.; Stoichkov, V.; Meroni, S. M. P.; Pockett, A.; Worsley, C. A.; Carnie, M.; Worsley, D. A.;

Watson, T. Enhancing Fully Printable Mesoscopic Perovskite Solar Cell Performance Using Integrated Metallic Grids to Improve Carbon Electrode Conductivity. *Curr. Appl. Phys.* **2020**, *20* (5), 619–627.

(24) Su, H.; Xiao, J.; Li, Q.; Peng, C.; Zhang, X.; Mao, C.; Yao, Q.; Lu, Y.; Ku, Z.; Zhong, J.; Li, W.; Peng, Y.; Huang, F.; Cheng, Y. Carbon Film Electrode Based Square-Centimeter Scale Planar Perovskite Solar Cells Exceeding 17% Efficiency. *Mater. Sci. Semicond. Process.* **2020**, *107*, 104809.

(25) Meng, F.; Gao, L.; Yan, Y.; Cao, J.; Wang, N.; Wang, T.; Ma, T. Ultra-Low-Cost Coal-Based Carbon Electrodes with Seamless Interfacial Contact for Effective Sandwich-Structured Perovskite Solar Cells. *Carbon* **2019**, *145*, 290–296.

(26) Cai, L.; Liang, L.; Wu, J.; Ding, B.; Gao, L.; Fan, B. Large Area Perovskite Solar Cell Module. *J. Semicond.* **2017**, *38* (1), 014006.

(27) Hu, Y.; Si, S.; Mei, A.; Rong, Y.; Liu, H.; Li, X.; Han, H. Stable Large-Area (10×10 cm^2) Printable Mesoscopic Perovskite Module Exceeding 10% Efficiency. *Sol. RRL* **2017**, *1* (2), 1600019.

(28) De Rossi, F.; Baker, J. A.; Beynon, D.; Hooper, K. E. A.; Meroni, S. M. P.; Williams, D.; Wei, Z.; Yasin, A.; Charbonneau, C.; Jewell, E. H.; Watson, T. M. All Printable Perovskite Solar Modules with 198 Cm 2 Active Area and Over 6% Efficiency. *Adv. Mater. Technol.* **2018**, *3* (11), 1800156.

(29) Meroni, S. M. P.; Hooper, K. E. A.; Dunlop, T.; Baker, J. A.; Worsley, D.; Charbonneau, C.; Watson, T. M. Scribing Method for Carbon Perovskite Solar Modules. *Energies* **2020**, *13* (7), 1589.

(30) Priyadarshi, A.; Haur, L. J.; Murray, P.; Fu, D.; Kulkarni, S.; Xing, G.; Sum, T. C.; Mathews, N.; Mhaisalkar, S. G. A Large Area (70 Cm^2) Monolithic Perovskite Solar Module with a High Efficiency and Stability. *Energy Environ. Sci.* **2016**, *9* (12), 3687–3692.
